# Conserved Motifs within Hepatitis C Virus Envelope (E2) RNA and Protein Independently Inhibit T Cell Activation

**DOI:** 10.1371/journal.ppat.1005183

**Published:** 2015-09-30

**Authors:** Nirjal Bhattarai, James H. McLinden, Jinhua Xiang, Thomas M. Kaufman, Jack T. Stapleton

**Affiliations:** 1 Research Service and, The Iowa City Veterans Affairs Medical Center, Iowa City, Iowa, United States of America; 2 The Departments of Internal Medicine and Microbiology, University of Iowa, Iowa City, Iowa, United States of America; The Scripps Research Institute, UNITED STATES

## Abstract

T cell receptor (TCR) signaling is required for T-cell activation, proliferation, differentiation, and effector function. Hepatitis C virus (HCV) infection is associated with impaired T-cell function leading to persistent viremia, delayed and inconsistent antibody responses, and mild immune dysfunction. Although multiple factors appear to contribute to T-cell dysfunction, a role for HCV particles in this process has not been identified. Here, we show that incubation of primary human CD4+ and CD8+ T-cells with HCV RNA-containing serum, HCV-RNA containing extracellular vesicles (EVs), cell culture derived HCV particles (HCVcc) and HCV envelope pseudotyped retrovirus particles (HCVpp) inhibited TCR-mediated signaling. Since HCVpp’s contain only E1 and E2, we examined the effect of HCV E2 on TCR signaling pathways. HCV E2 expression recapitulated HCV particle-induced TCR inhibition. A highly conserved, 51 nucleotide (nt) RNA sequence was sufficient to inhibit TCR signaling. Cells expressing the HCV E2 coding RNA contained a short, virus-derived RNA predicted to be a Dicer substrate, which targeted a phosphatase involved in Src-kinase signaling (PTPRE). T-cells and hepatocytes containing HCV E2 RNA had reduced PTPRE protein levels. Mutation of 6 nts abolished the predicted Dicer interactions and restored PTPRE expression and proximal TCR signaling. HCV RNA did not inhibit distal TCR signaling induced by PMA and Ionomycin; however, HCV E2 protein inhibited distal TCR signaling. This inhibition required lymphocyte-specific tyrosine kinase (Lck). Lck phosphorylated HCV E2 at a conserved tyrosine (Y613), and phospho-E2 inhibited nuclear translocation of NFAT. Mutation of Y613 restored distal TCR signaling, even in the context of HCVpps. Thus, HCV particles delivered viral RNA and E2 protein to T-cells, and these inhibited proximal and distal TCR signaling respectively. These effects of HCV particles likely aid in establishing infection and contribute to viral persistence.

## Author Summary

Globally, approximately 200 million people are persistently infected with Hepatitis C virus (HCV). Mechanisms by which HCV establishes persistent infection are complex, and several host and viral factors appear to contribute to the ability of HCV to evade immune clearance. T cell activation through the T cell receptor (TCR) is an essential first step in the generation of an adaptive immune response. Although HCV infection is associated with impaired T cell function, the mechanisms for this dysfunction are poorly understood. Here, we demonstrate that HCV particles inhibit T cell activation by interfering with proximal and distal signals that are triggered by activation through the TCR. First, HCV envelope (E2) RNA was processed into a small RNA that targeted a regulatory phosphatase, inhibiting proximal TCR signaling. Second, the lymphocyte specific Src kinase (Lck) phosphorylated HCV E2 at tyrosine 613 (Y613), and phospho-E2 inhibited nuclear translocation of activated NFAT, reducing distal TCR activation signals. The RNA and protein motifs involved are highly conserved among all HCV isolates, and mutation restored TCR signaling. Thus, HCV particles interfere with TCR signaling and impair T cell activation using two distinct mechanisms. This may contribute to HCV persistence and T cell dysfunction during HCV infection.

## Introduction

Hepatitis C virus (HCV) infects more than 180 million people worldwide and is a leading cause of liver disease [1]. Most infected individuals develop chronic viremia which frequently leads to cirrhosis and hepatocellular carcinoma [1,2]. Hallmarks of HCV infection include impaired HCV-specific intrahepatic and peripheral T cell responses and a delayed onset of HCV-specific humoral and cellular immunity [3–11]. HCV infection is also associated with impaired immune response against vaccine antigens like HBV and reduction in organ transplant rejection, suggesting a state of mild general immunosuppression [12–14]. Although previous studies found that T cell activation is reduced in HCV-infected people [3–11], mechanisms explaining this immune impairment are not well characterized.

HCV replicates primarily in hepatocytes, and infected cells secrete viral particles and extracellular vesicles (EV) containing viral RNA and envelope proteins [15–18]. Secreted EVs are capable of interacting with and modulating immune functions [18–20]. HCV RNA is also found in patient peripheral blood mononuclear cells (PBMCs) [21–28]. Due to high serum concentrations of HCV in infected individuals, abundant interactions occur between HCV particles and lymphocytes.

T cell receptor (TCR) signaling is required for activation, proliferation, and effector function of CD4+ and CD8+ T cells, and is critical for inducing an effective immune response [29]. Although numerous factors are proposed to contribute to viral persistence and the ineffective host immune response to HCV infection [30,31], none of these fully explain the weak and ineffective induction or maintenance of T cell responses to HCV proteins in infected individuals. Here, we assessed the effect of HCV particles, E2, and RNA on T cell activation. Two novel and distinct mechanisms of inhibition of TCR signaling were found, and both were mediated by conserved HCV E2 sequences.

## Results

### HCV particles inhibit TCR signaling in primary human T cells

**Fig 1:**
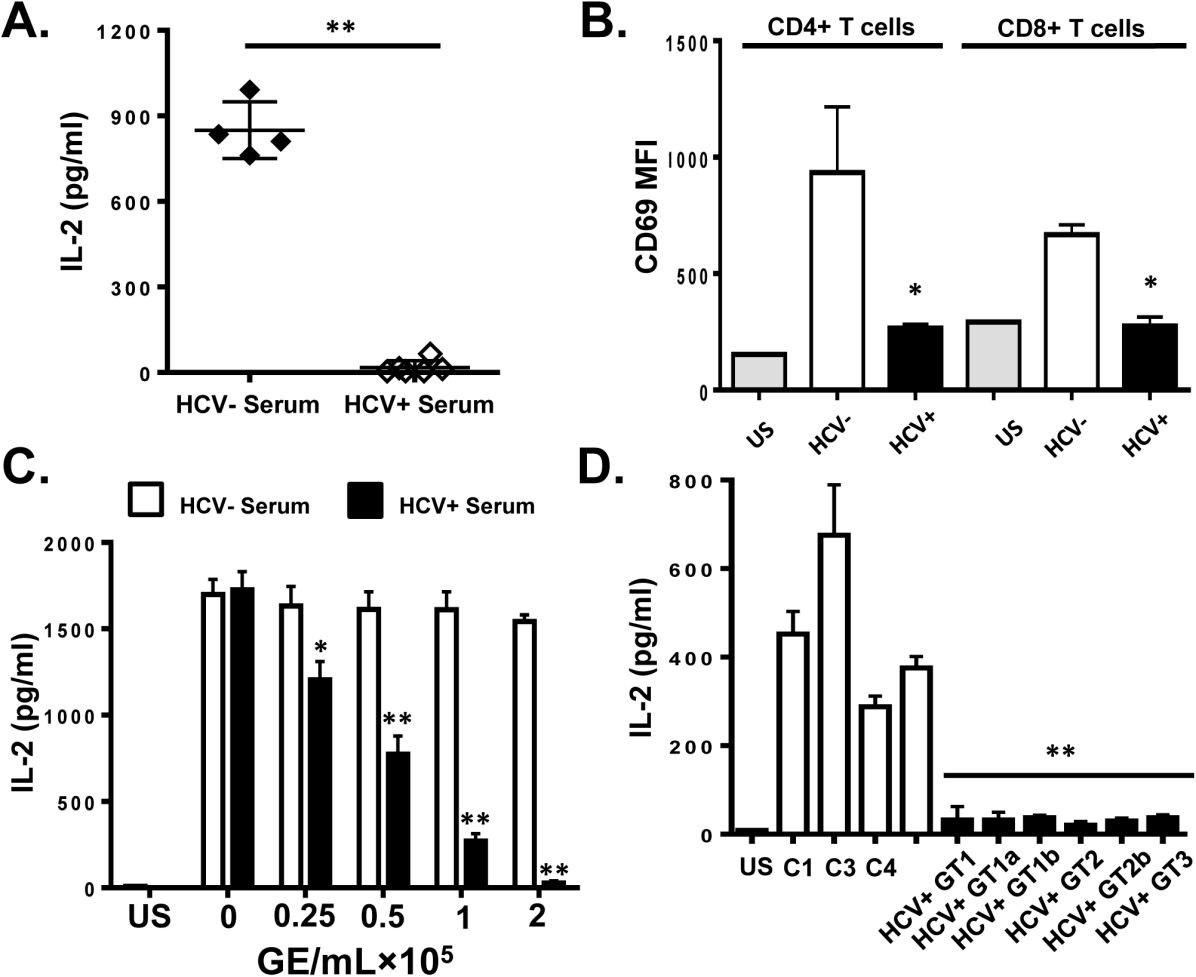
HCV serum particles inhibit T cell receptor (TCR) signaling in primary human T lymphocytes. Healthy donor PBMCs were incubated with serum obtained from HCV positive (HCV+) humans infected with various genotypes and subtypes (GT; 1, 1a, 1b, 2, 2b, and 3) or HCV negative control serum (C1-C4) and IL-2 release (A) and CD69 surface expression (B) were measured following TCR stimulation with anti-CD3/CD28. CD69 MFI represents the average of four HCV negative and six HCV-positive sera samples. TCR-induced IL-2 release by human PBMCs incubated with various doses of pooled HCV positive or HCV negative serum (C). IL-2 release by purified primary human CD3+ T cells incubated with HCV-positive sera from genotypes (GT; 1, 1a, 1b, 2, 2b, and 3) or HCV negative serum (C1-C4) (D). US = unstimulated cells. MFI = mean fluorescent intensity. Data represent the average of three technical replicates and the standard deviation is shown. Each experiment was independently performed with three different donors with similar results. *P< 0.05; **P< 0.01.

Serum from HCV-infected subjects (genotypes 1, 1a, 1b, 2, 2b and 3; RNA concentration range 1 x 10^5^ to 1 x 10^6^ RNA genome equivalents [GE] per mL), or HCV uninfected controls were incubated with PBMCs obtained from healthy blood donors. Following TCR stimulation with anti-CD3/CD28, signaling was quantified by measuring IL-2 release into culture supernatants or by measuring CD69 expression on these primary CD4+ and CD8+ T lymphocytes. All HCV RNA positive sera inhibited IL-2 release (Fig 1A) and CD69 surface expression (Fig 1B) in a dose-dependent manner (Fig 1C) compared to HCV-uninfected controls. To ensure that this inhibition was not due to interactions with non-T cells within the PBMCs, T cells were purified (>99% pure, S1 Fig) and HCV RNA positive sera again inhibited T cell activation as measured by IL-2 release (Fig 1D).

**Fig 2:**
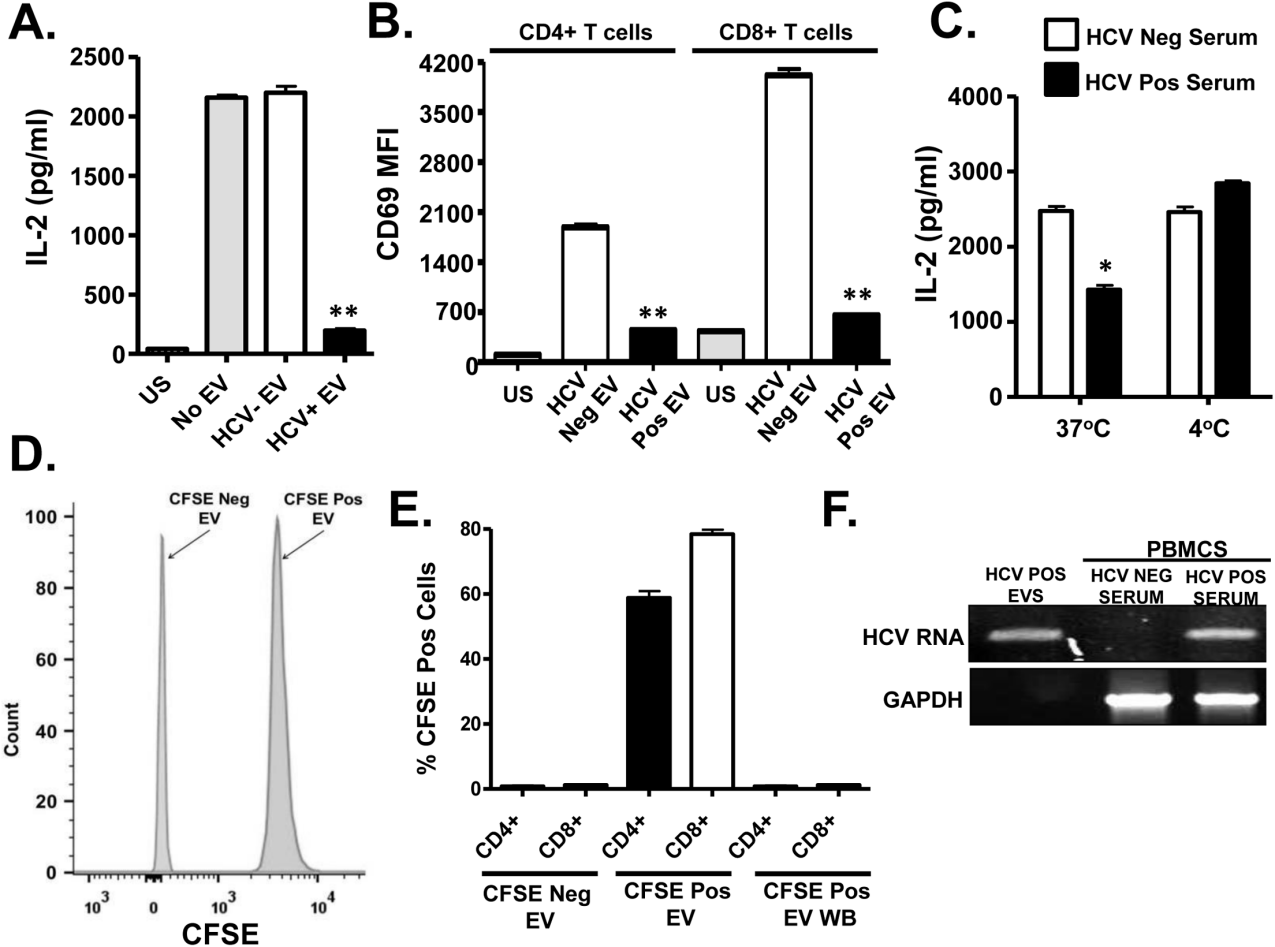
HCV serum derived extracellular vesicles (EV) inhibit T cell receptor (TCR) signaling in primary human T lymphocytes. Healthy donor PBMCs were incubated with pooled serum extracellular vesicles (EV) obtained from HCV-infected patient sera (HCV+ EV; GT 1, 1a, 1b, 2, 2b and 3). TCR induced IL-2 (A) and CD69 surface expression (B) was measured. TCR induced IL-2 release by human T cells incubated with HCV-positive or negative serum for two hours at 37°C or 4°C (C). Analysis of CFSE positive serum EV (D) and uptake of CFSE-positive EV by primary human T cells (E) as determined by flow cytometry. HCV RNA was detected using RT-PCR in EVs from HCV-positive serum, and in human PBMCs incubated with HCV-positive but not HCV RNA negative serum (F). HCV RNA US = unstimulated cells. MFI = mean fluorescent intensity. Data represent the average of three technical replicates, and the standard deviation is shown. Each experiment was independently performed with three different donors with similar results. *P< 0.05; **P< 0.01.

To remove serum factors that may interfere with TCR signaling, serum extracellular vesicles (EV) were prepared using a commercial reagent (Exoquick, Systems Biosciences). This method of purification yields well characterized EV consistent with exosomes from human serum [32]. Exosomes are reported to contain HCV envelope proteins, HCV RNA and to transmit HCV infection *in vitro* [15–19]. Purified HCV RNA-containing EVs contained CD63 and CD81 but did not contain CD69 and CD25 (S2A Fig), supporting an endocytic source of origin. Incubation of PBMCs with EV from HCV-infected individuals inhibited IL-2 release and CD69 expression (Fig 2A and 2B, respectively) compared to HCV-negative control EV. Although this method of preparation yields EVs with characteristics of exosomes, we cannot exclude the possibility that some viral particles are included in the preparation.

To further examine EV interactions with lymphocytes, sera from HCV infected or uninfected subjects were incubated with the purified T cells obtained from 2 healthy blood donors at 37°C or at 4°C. Following two hour incubation and wash (room temperature), cells were stimulated with anti-CD3/CD28. HCV positive sera significantly inhibited TCR signaling at 37°C but not 4°C (Fig 2C). The inhibition was lower in magnitude, presumably due to the shortened incubation compared to earlier experiments. To assess EV fusion with cells, serum EVs from HCV positive or negative subjects were labeled with carboxy fluorescein succinimidyl ester (CFSE) (Fig 2D) prior to incubation with primary human PBMCs. EV transferred CFSE to both CD4+ and CD8+ T cells during overnight incubation (Fig 2E). Since CFSE is a cell-permeable dye and trace amounts of CFSE could lead to a positive result, cells were also incubated in the final wash buffer (EV wash). CFSE was not detected in these cells (Fig 2E). Further, HCV RNA was detected in PBMCs following incubation with EVs, thus EVs transferred viral RNA to PBMCs during incubation (Fig 2F). In summary, serum-derived HCV particles fused with and transferred viral RNA into T cells. To exclude the possibility that serum factors mediated uptake of virus or EV's by T cells *in vitro*, HCV RNA was amplified from purified T cells (>97% pure) as described above. HCV RNA was present in both PBMCs and purified T cells obtained from HCV-infected subjects, whereas viral RNA was not detected in HCV-uninfected subject (S2B Fig and S2C Fig).

**Fig 3:**
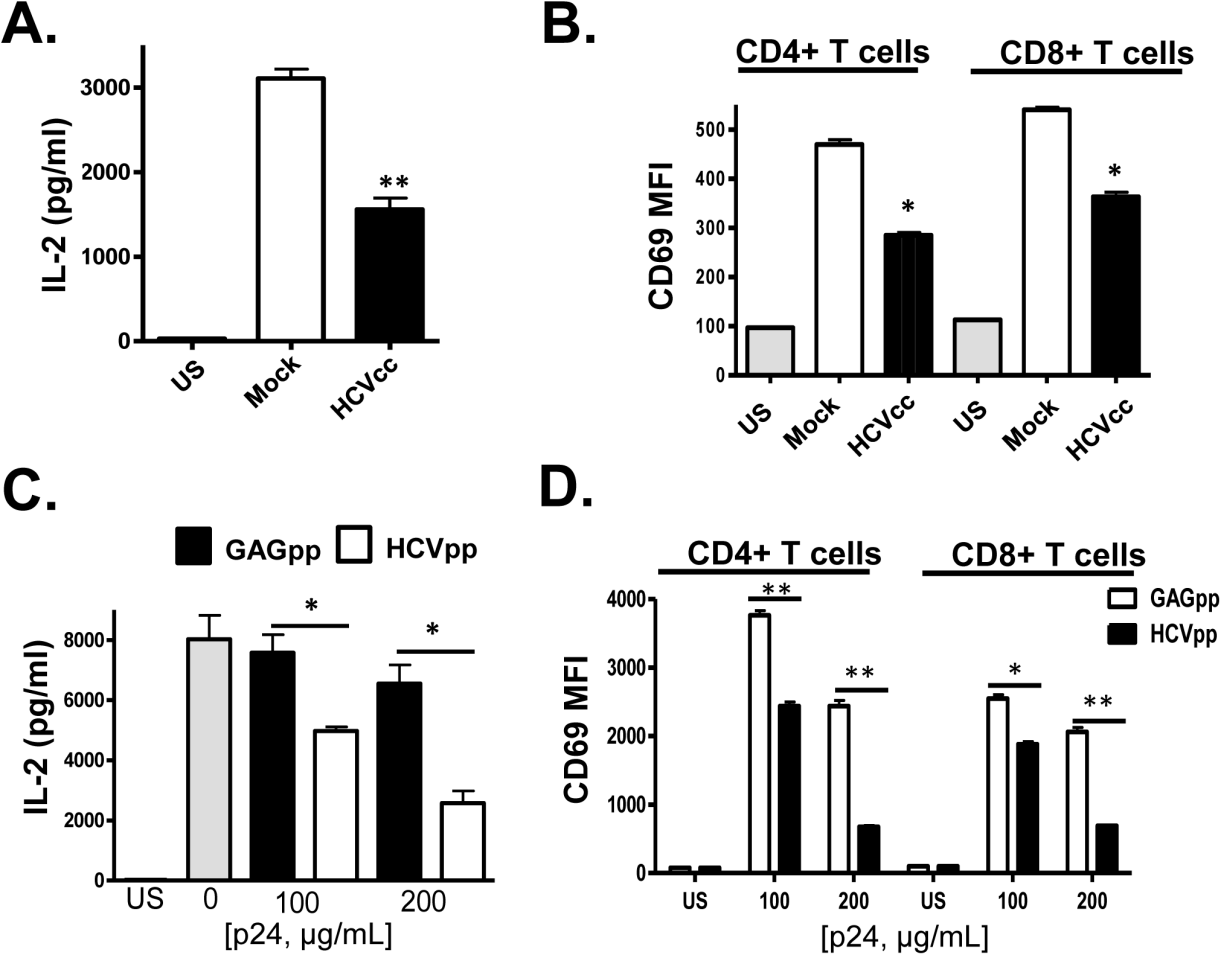
HCV cell culture derived particles (HCVcc) and HCV envelope pseudotyped retrovirus particles (HCVpp) inhibit T cell receptor (TCR) signaling in primary human T cells. HCVcc produced in Huh7.5 cells inhibited TCR-mediated IL-2 release (A) and CD69 surface expression (B) in human peripheral blood mononuclear cells compared to cells incubated in mock-transfected Huh7.5 cell culture supernatant fluids following TCR stimulation with anti-CD3/CD28. Similarly, HCVpp’s inhibited TCR-mediated IL-2 release (C) and CD69 surface expression (D) compared to cells incubated in retrovirus GAG particles in a dose-related manner. US = unstimulated cells. MFI = mean fluorescent intensity. Data represent the average of three technical replicates, and the standard deviation is shown. Each experiment was independently performed with three different donors with similar results. *P< 0.05; **P< 0.01.

Next, infectious HCV particles generated in a hepatocyte cell line (Huh7.5 cells) were incubated with PBMCs prior to TCR stimulation. Similar to serum-derived EV, cell culture infectious HCV particles (HCVcc) inhibited TCR signaling in CD4+ and CD8+ T cells (Fig 3A and 3B). Viral replication was not required, as replication defective retrovirus particles pseudotyped with the HCV E1 and E2 (HCVpp) also inhibited TCR signaling in a dose-dependent manner compared to non-enveloped retrovirus particles (GAGpp; Fig 3C and 3D). To ensure that the HCV particles inhibited IL-2 release following TCR stimulation, purified T cells obtained from two healthy blood donors (>99% pure as in S1 Fig) were incubated with HCVcc particles or HCVpps prior to stimulation. Both particle types inhibited TCR signaling in these purified CD3+ T cells (S3 Fig). Thus, TCR signaling inhibition is mediated by HCV particles in the absence of serum or non-T cells within PBMCs.

In summary, incubation of primary human PBMCs or purified T cells with i) serum from HCV-infected individuals, ii) HCV RNA and CD63/CD81 containing serum-derived EV, iii) HCVcc and iv) HCVpp reduced TCR-mediated activation in primary human CD4+ and CD8+ T cells compared to controls.

### HCV E2 coding RNA inhibits proximal TCR signaling pathways

**Fig 4:**
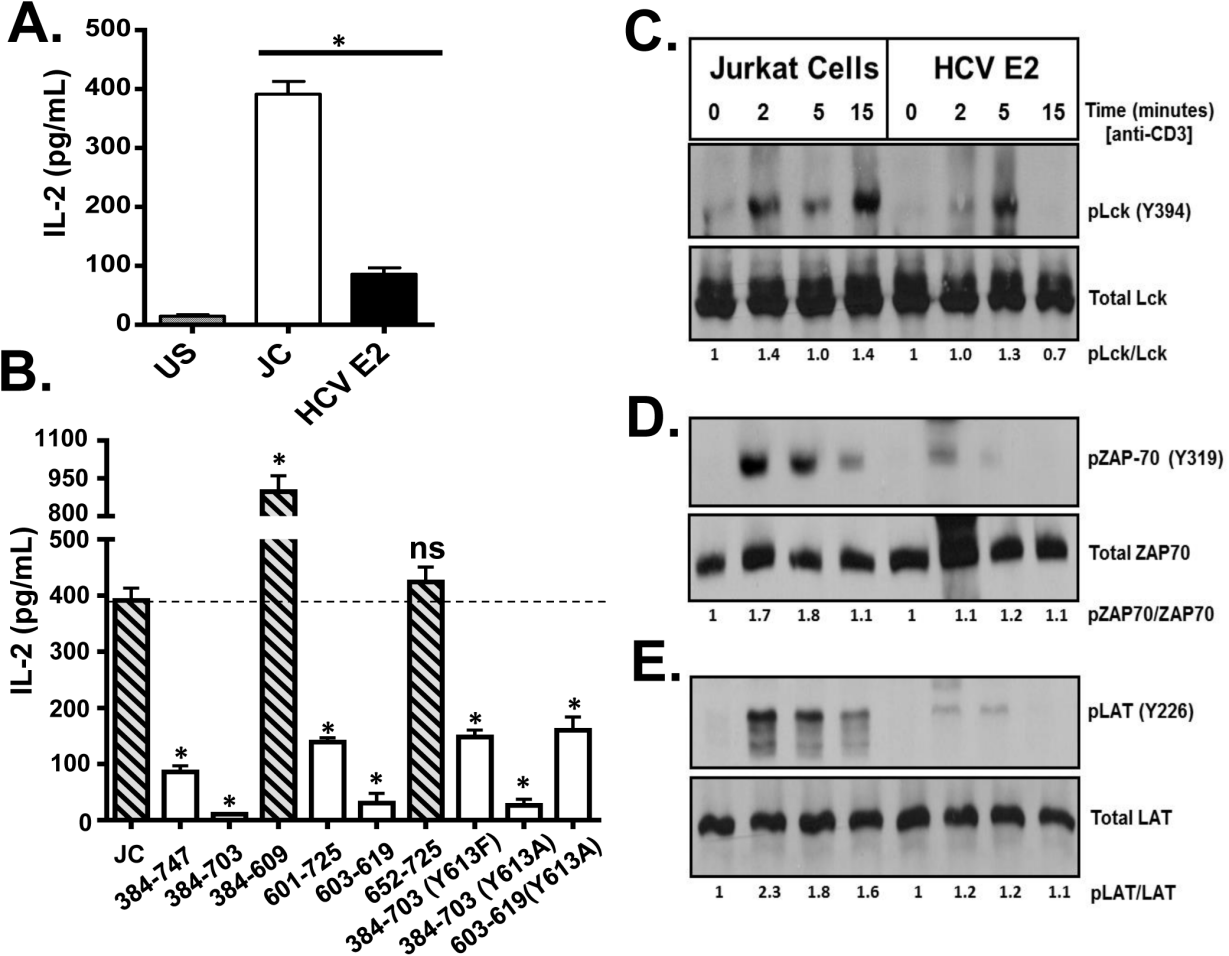
HCV envelope protein E2 inhibits T cell receptor (TCR)-mediated signaling. Jurkat control cells (JC) or Jurkat cells stably expressing HCV E2 protein were stimulated with anti-CD3 and anti-CD28. Twenty-four hours later, IL-2 release (A) was measured. Phosphorylation and activation of the lymphocyte specific tyrosine kinase (Lck Y394; B), the zeta-chain-associated protein kinase (ZAP)-70 (Y319; C) and linker for activation of T cells (LAT, Y226; D) was analyzed in HCV E2 expressing Jurkat cells compared to the controls (JC) following TCR activation using anti-CD3. IL-2 released in truncated or substitution mutant HCV E2 proteins expressing Jurkat cells are shown in panel E. The amino acid numbers relate to their location on the HCV polyprotein. Phospho-blots for Lck, ZAP-70 and LAT were performed at least three times with consistent results. Data represent the average of three technical replicates and the standard deviation is shown. Each experiment was independently performed three times with consistent results. *P< 0.01, ns = not significant.

Since HCVpp particles only contain HCV E1 and E2, we next examined the major HCV envelope glycoprotein (E2) for its ability to inhibit T cell activation through the TCR. Since HCV does not replicate well, if at all, in lymphocytes, we generated Jurkat (CD4+) T cell lines stably expressing HCV E2 protein (S4A Fig). In the cells expressing full-length E2 (aa 384–747), there was significantly less TCR-mediated activation as determined by IL-2 release (Fig 4A) or cell surface CD69 expression (S4B Fig) when compared to control Jurkat cells expressing only GFP. To characterize HCV E2 region(s) required for inhibition of TCR signaling, a series of Jurkat cell lines expressing truncated E2 proteins were generated (S4C Fig). HCV E2 expression in individual cell lines was comparable (S5A Fig). IL-2 release following TCR stimulation was reduced in all cell lines expressing an E2 fragment containing aa 603 to 619 (Fig 4B). In contrast, IL-2 release was not inhibited in cells expressing HCV E2 protein that did not contain this region.

The most proximal kinase in the TCR signaling cascade is the lymphocyte-specific protein tyrosine kinase (Lck) [33]. Inactive Lck is phosphorylated at tyrosine 505 (Y505) by the c-src tyrosine kinase (Csk). Following TCR engagement, Y505 is dephosphorylated by many tyrosine phosphatases including CD45, resulting in conformational changes and subsequent auto-*trans*-phosphorylation at tyrosine 394 (Y394). Phosphorylated Lck (Y394) is the active kinase required for subsequent downstream signaling. Following TCR stimulation, Lck phosphorylation (Y394) was reduced in Jurkat cells expressing HCV E2 protein compared to controls (Fig 4C). Activated Lck is required for activation of both zeta-chain-associated protein kinase (ZAP)-70 and the linker for activation of T cells (LAT). Consistent with reduced Lck activation, ZAP-70 and LAT phosphorylation were reduced in HCV E2 expressing cells compared to controls (Fig 4D and 4E).

Using kinase-specific phosphorylation substrate prediction models, the tyrosine at HCV E2 aa 613 (Y613) was predicted to be a Src/Lck substrate [34]. This region (aa 603–619) is highly conserved and the Y613 is conserved in more than 600 isolates representing all HCV genotypes (http://www.hcv.lanl.gov). Previous studies found that a conserved tyrosine in the related human Pegivirus (HPgV) is required TCR-signaling inhibition, and mutation of this residue restores TCR signaling [35].

Thus, HCV E2 Y613 was mutated to alanine (Y613A) in the context of the peptide (HCV aa 603–619), or to alanine or phenylalanine (Y613A, Y613F) in the context of the E2 protein with the C-terminal transmembrane domain truncated (S5B Fig and S5C Fig). Y613 mutation did not restore TCR signaling following TCR stimulation (Fig 4B). Iinhibition of IL-2 release by HCV E2 expression was not due reduced CD45 and Csk expression levels, as they were similar in HCV E2 expressing cells and control cells (S6A Fig and S6B Fig, respectively).

**Fig 5:**
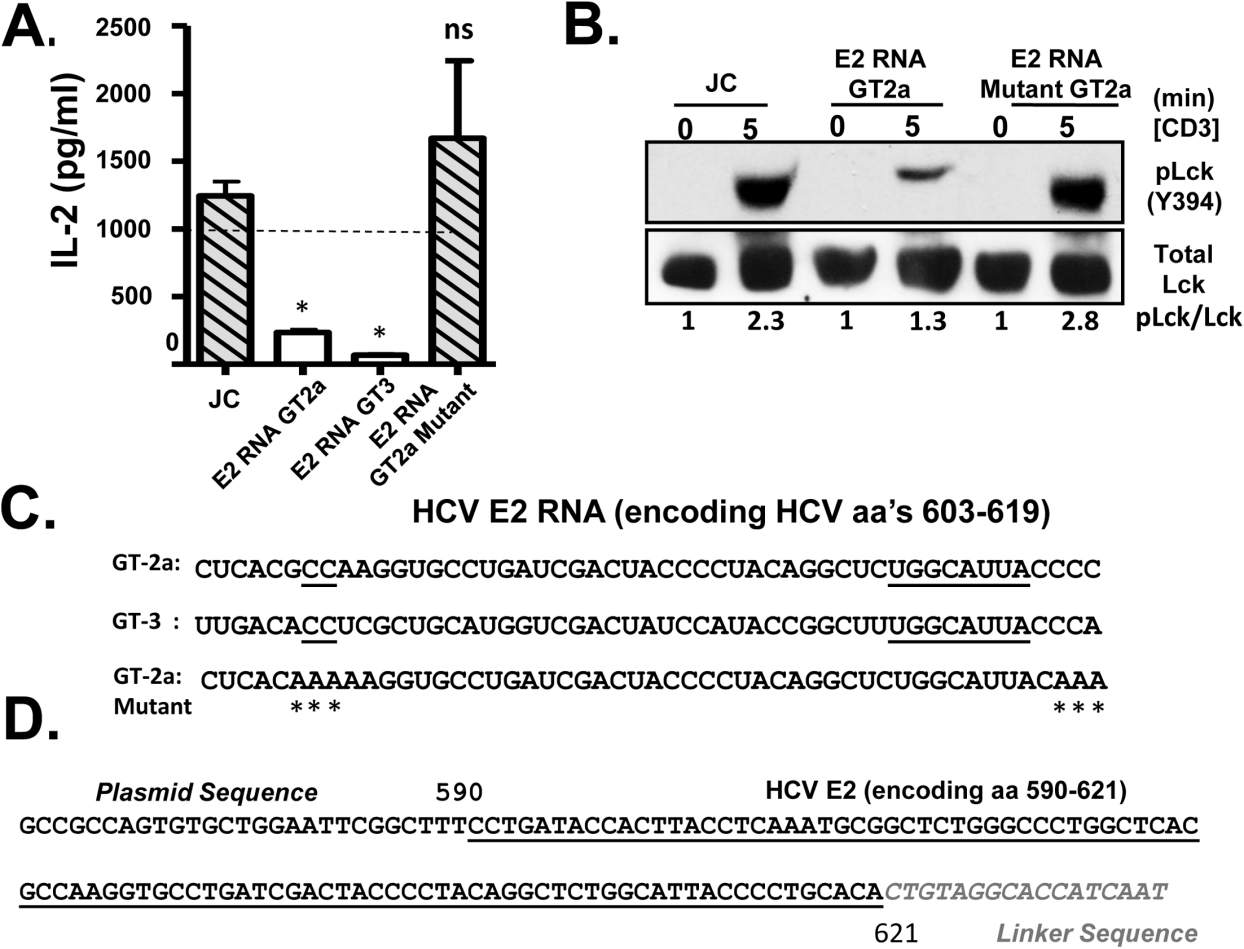
HCV envelope (E2) coding RNA is sufficient to inhibit proximal T cell receptor (TCR) signaling. Jurkat cells were generated that stably expressed HCV envelope (E2) RNA (coding aa 384–703) with a frame-shift mutation to abolish protein expression from isolates belonging to genotype (GT) 2a and GT3, or the GT 2a sequence in which four cytidine residues were changed to alanine residues. TCR induced IL-2 release from these Jurkat cells were measured after 24 hour stimulation with anti-CD3/CD28 (A). Activation of lymphocyte specific tyrosine kinase (Lck) was measured by immunoblotting for phosphoY394 following anti-CD3 stimulation (B). Total Lck served as the loading control. The RNA sequence of the HCV E2 (aa 603–619) coding region from the different HCV genotypes (GT) and mutants are shown in panel (C). Conserved sequences are underlined and mutations introduced into the GT 2a sequence noted by * (C). Small RNAs were amplified following 3’linker ligation and specific cDNA synthesis. Small RNAs were cloned and sequenced, and the HCV E2 region encoding (aa 590–621) was detected in Jurkat cells expressing HCV E2 protein. Panel (D) demonstrates the partial sequence of the plasmid (pCR2.1) and HCV E2 RNA amplification product, followed by the oligonucleotide linker sequence. *P< 0.01; ns = not significant.

To determine if E2 protein was required for TCR inhibition, a Jurkat cell line expressing HCV E2 RNA coding sequences with a frame-shift mutation was generated. This cell line expressed HCV E2 RNA, but not E2 protein (S5A Fig and S5C Fig). To our surprise, expression HCV E2 RNA was sufficient to inhibit TCR signaling as measured by both IL-2 release (Fig 5A) and Lck phosphorylation (Fig 5B). Thus, E2 RNA encoding aa 603–619 was required and sufficient for inhibition of T cell activation mediated by TCR engagement.

HCV has considerable sequence diversity among isolates, including the sequences encoding E2 aa 603–619 (http://www.hcv.lanl.gov). The HCV E2 RNA and protein expression from a different HCV isolate (genotype 3; GT-3), containing 13 nt differences in the RNA coding aa 603–619 were examined (Fig 5C). Like GT-2a, GT-3 E2 RNA inhibited TCR-mediated IL-2 release (Fig 5A). Despite some sequence diversity, 4 cytosine residues are conserved in more than 600 HCV isolates representing all genotypes (Fig 5C). We generated a Jurkat cell line expressing a HCV E2 RNA with the cytosine residues mutated to adenosine (Fig 5C), and TCR signaling was restored in cells expressing this mutation as measured by IL-2 release and phosphorylation of Lck following anti-CD3/CD28 (Fig 5A and 5B).

Bioinformatics analyses predicted that the conserved nucleotides within the HCV E2 603–619 coding RNA sequences generate an RNA structure that would be processed by Dicer, the cytoplasmic endoribonuclease involved in the microRNA (miRNA) pathway (S7 Fig) [36]. Mutation of the conserved cytosines that rescued TCR signaling resulted into RNA structure that did not fold into a Dicer substrate (S7 Fig). Previous studies identified interactions between HCV RNA and miRNA pathway including Dicer [37,38], and HCV virus-derived, small RNAs (vd-sRNAs) are found in HCV infected cells, including RNAs from the E2 coding region [39]. To determine if vd-sRNAs were present in E2 expressing cells, total cellular RNA was analyzed for the presence of small, E2 derived RNAs as described in the Methods. Following amplification and sequence analysis of RNA species present in these cells, a vd-sRNA containing the T cell inhibitory RNA region encoding HCV E2 aa 590–621 was identified (Fig 5D). Thus, full length HCV E2 RNA was processed into TCR inhibitory vd-sRNAs in these cells.

**Fig 6:**
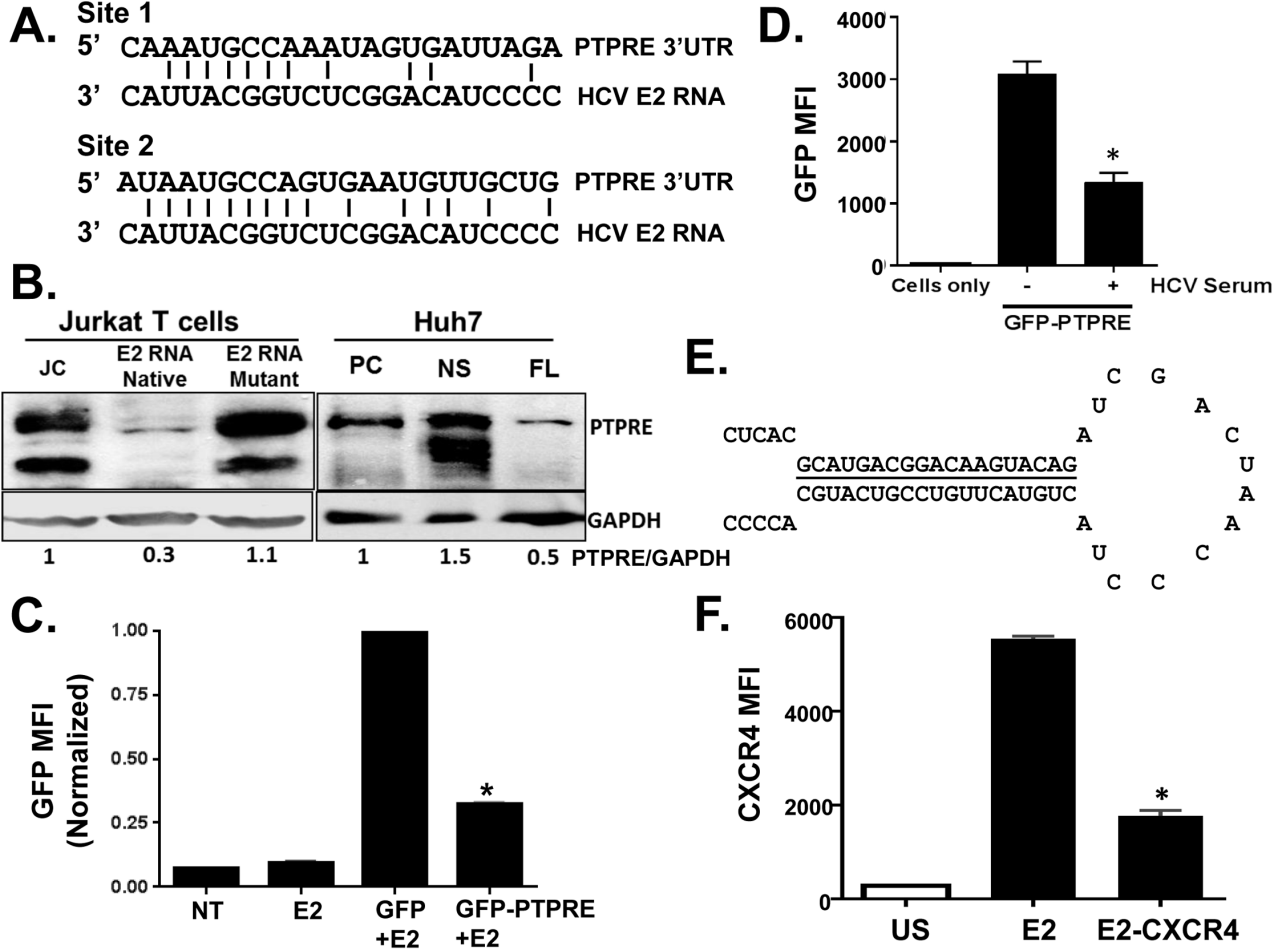
HCV E2 RNA inhibits protein tyrosine phosphatase receptor type E (PTPRE) expression. Sequence alignment of two sites within PTPRE 3’ untranslated region (UTR) predicted to bind to HCV E2 RNA (aa 603–619) region (A). Immunoblot analysis of PTPRE protein levels in control, HCV E2 RNA or HCV E2 mutant RNA expressing Jurkat cells, or Huh7 cells expressing full length HCV replicon (FL) or non-structural protein (NS) expressing replicon. The upper band represents full-length PTPRE with transmembrane domain (isoform-1) and lower band represents cytoplasmic PTPRE (isoform-2). GAPDH serves as a loading control (B). GFP expression by HEK 293 cells co-transfected with 1μg of plasmid DNA encoding GFP alone or GFP with PTPRE 3’UTR sequence shown in panel A and 5 μg of plasmid DNA encoding HCV E2 (C) or incubated with HCV-positive serum (D) and GFP expression measured after 72 hours. Data represent the average of three technical replicates and each experiment was repeated at least three times with consistent results. The region of HCV E2 targeting PTPRE was replaced with sequences targeting the cellular chemokine receptor CXCR4 (E), and a Jurkat cell line stably expressing this sequence was generated. CXCR4 was reduced in Jurkat cells expressing this HCV E2 sequence targeting CXCR4, but not Jurkat cells expressing the native HCV E2 RNA sequence (F). *P<0.01.

To understand the mechanism by which vd-sRNA inhibits TCR signaling, additional analyses were performed to identify potential targets for this vd-sRNA sequence. Two conserved sites complementarity to vd-sRNA were found within the 3’ untranslated region (UTR) of a protein tyrosine phosphatase type E (PTPRE; Fig 6A). PTPRE regulates Src family kinases, of which Lck is a member [40–44]. PTPRE mRNA expression levels were similar in control and HCV E2 RNA expressing cells (S8 Fig); however, Jurkat cells expressing E2 RNA had significantly reduced PTPRE protein levels compared to controls (Fig 6B). The upper band represents the full-length PTPRE with a transmembrane domain (isoform 1) while the lower band represents cytoplasmic PTPRE (isoform 2) (Fig 6B). Mutation of the conserved nucleotides in E2 RNA to remove the predicted Dicer substrates restored PTPRE protein expression (Fig 6B) and TCR signaling (Fig 5A and 5B). PTPRE protein levels were also reduced in human hepatoma (Huh) cells containing full length HCV RNA in replicons (FL) compared to parent Huh cells or Huh7 containing HCV replicons expressing only nonstructural proteins (NS) (Fig 6B).

To determine the specificity and HCV E2 coding RNA requirements for PTPRE knockdown, we inserted the PTPRE 3’UTR sequence into the 3’UTR of GFP in an expression plasmid. GFP expression in 293T cells was reduced by co-transfection of HCV E2 coding plasmid compared to GFP without PTPRE 3’UTR (Fig 6C). Furthermore, incubation of 293T cells in HCV RNA containing serum reduced GFP expression compared to cells incubated in control (HCV RNA negative) serum (Fig 6D). Thus, HCV RNA encoding envelope E2 directly targets PTPRE and inhibits its expression.

To further examine the specificity of the HCV E2 RNA for targeting cellular genes, we replaced the predicted seed sequence for PTPRE with a sequence targeting a cellular gene expressed in Jurkat cells (CXCR4) (Fig 6E). A Jurkat cell line was generated and CXCR4 expression was examined. Replacing PTPRE targeting sequence with CXCR4 significantly reduced CXCR4 expression (Fig 6F).

Together, these data demonstrate that HCV E2 RNA expressed *in vitro* is processed into short RNA that inhibits PTPRE expression in human hepatocyte (Huh 7) and T (Jurkat) cells, and inhibits TCR-mediated Src (Lck) signaling. Addition of HCV RNA-containing serum to 293 cells also inhibits PTPRE expression, thus this effect is highly likely to be biologically relevant.

### HCV E2 protein inhibits distal TCR signaling

**Fig 7:**
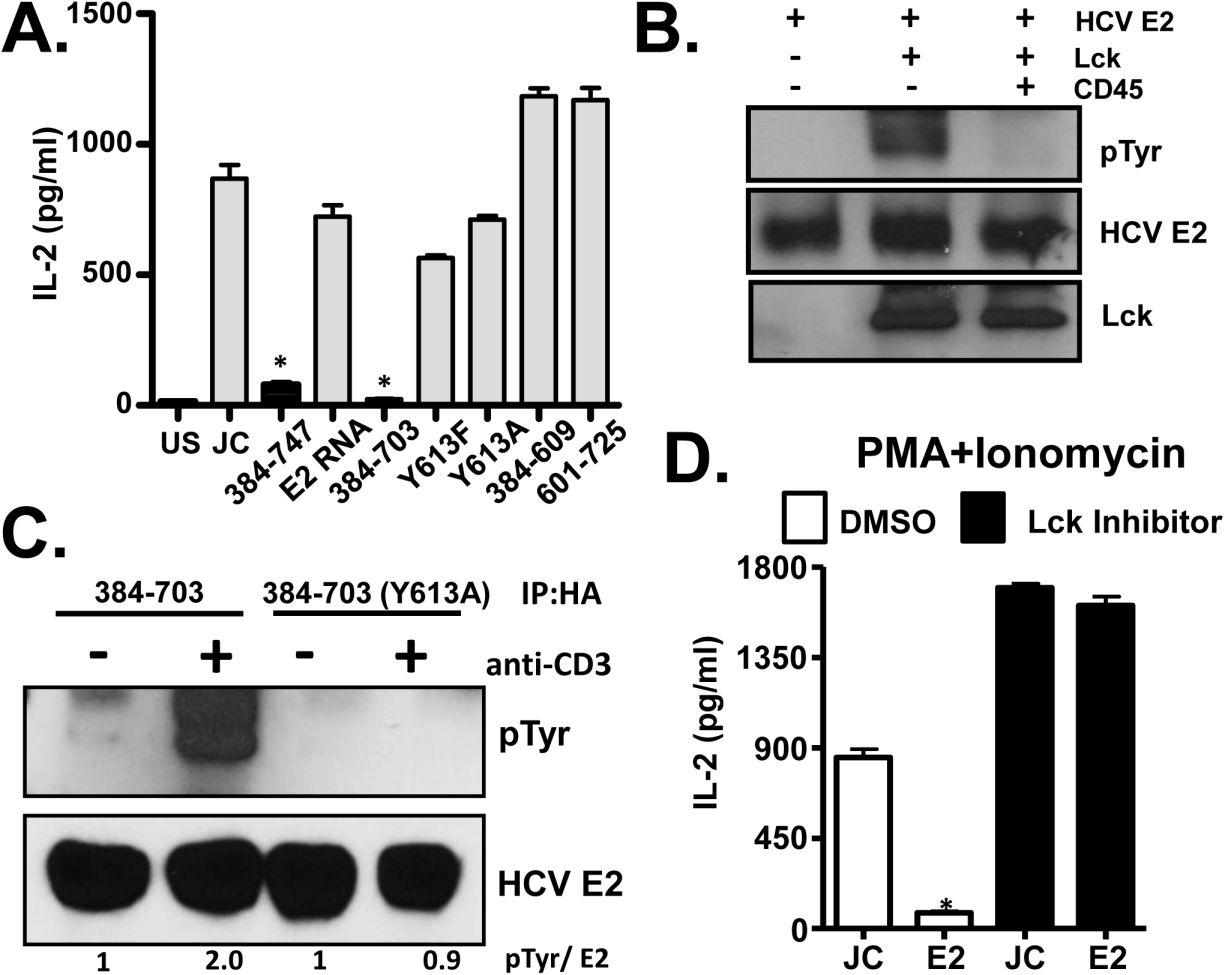
HCV E2 protein inhibits distal T cell receptor (TCR) signaling. PMA + Ionomycin (P+I) mediated IL-2 release by Jurkat cells expressing full-length or various truncated or tyrosine 613 mutant HCV E2 protein fragments as indicated (A). Recombinant HCV E2 protein was phosphorylated by Lck in an *in vitro* kinase reaction, and was dephosphorylated by the CD45 phosphatase (B). HCV E2 protein (native, or Y613A mutant) expressed in Jurkat cells was precipitated before (-) or after (+) TCR stimulation with anti-CD3. E2 and phospho-E2 were detected by immunoblot with E2 specific antibody or anti-phosphotyrosine antibody respectively (C). P+I mediated IL-2 release control Jurkat cells (JC) or HCV E2-expressing Jurkat cells (384–747) which had been incubated in 100μg/ml Lck inhibitor or the vehicle control (DMSO) (D). Data represent the average of three technical replicates and the standard deviation is shown. Each experiment was repeated at least three times with consistent results. *P< 0.01.

T cell activation can be initiated *in vitro* by stimulating downstream of TCR using phorbol-12-myristate-13-acetate (PMA) and ionomycin (P+I). To determine if HCV E2 RNA inhibited proximal and distal TCR-mediated signaling, Jurkat cells were stimulated with P+I, and cells expressing just HCV RNA did not inhibit distal signaling (Fig 7A). Thus, the viral RNA was specific for proximal signaling inhibition. To our surprise, HCV E2 protein expression with (aa 384–747), and without (aa 384–703) the transmembrane domain inhibited distal signaling following P+I activation (Fig 7A). Inhibition was specific for the NFAT pathway, as P+I stimulation did not inhibit CD69 expression in either HCV E2 RNA or E2 protein expressing cells (S9 Fig and S10 Fig). Near full length E2 (384–703) was required, as Jurkat cells expressing truncated E2 (384–609) or (601–725) did not inhibit distal signaling (Fig 7A). The conserved E2 Y613 was also required, as mutation of the predicted Lck substrate site (Y613F, Y613A) in the context of the near full-length protein restored P+I-mediated IL-2 release (Fig 7A).

The Y613 of HCV E2 protein is a predicted Lck substrate, thus phosphorylation of this residue was tested. *In vitro* recombinant HCV E2 was phosphorylated by Lck and dephosphorylated by CD45 (Fig 7B), and HCV E2 expressed in Jurkat cells was phosphorylated following TCR stimulation (Fig 7C). Thus, HCV E2 served as an Lck substrate and phosphorylation occurred at Y613, as the Y613A mutant was not phosphorylated following TCR engagement (Fig 7C). To assess the role of Lck mediated phosphorylation of HCV E2 in NFAT signaling, Jurkat cells were treated with Lck inhibitor overnight. P+I mediated IL-2 release was rescued in HCV E2 expressing cells treated with Lck inhibitor, suggesting that Lck-mediated phosphorylation of HCV E2 at Y613 was required to inhibit distal TCR signaling (Fig 7D). Together, these data identified a novel role of the T cell specific kinase Lck in phosphorylating the conserved tyrosine Y613 on HCV E2 for inhibition of E2 mediated distal TCR signaling.

**Fig 8:**
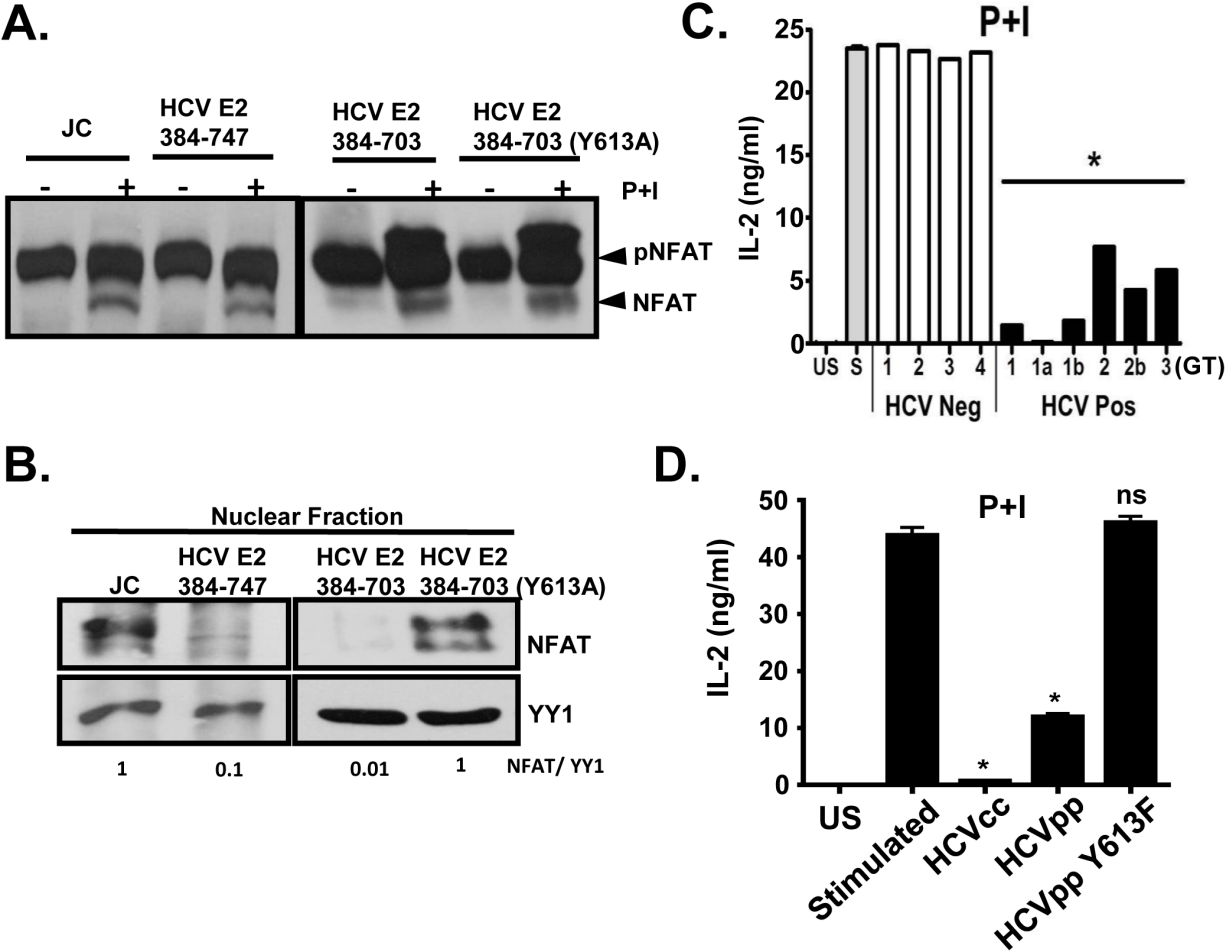
HCV E2 protein inhibits NFAT nuclear translocation. Dephosphorylation (A) and nuclear translocation (B) of the nuclear factor of activated T cells (NFAT) in Jurkat control cells or HCV E2 expressing cells as determined by immunoblot. The nuclear transcription factor Yin Yang 1 (YY1) served as the loading control for nuclear localization. P+I mediated IL-2 was release from primary healthy donor peripheral blood mononuclear cells (PBMCs) incubated with serum obtained from HCV positive (HCV+) humans infected with genotype (GT; 1, 1a, 1b, 2, 2b, and 3) HCV negative (HCV-) human subjects (C1-C4) (C) or cell-culture derived HCV particles (HCVcc) and retroviral particles pseudotyped with HCV envelope (E1-E2; HCVpps) or with HCV envelope containing the Y613F mutation (HCVpp Y613F) (D). US = unstimulated, and S = stimulated (no serum). Data represent the average of three technical replicates and the standard deviation is shown. Each experiment was repeated with three donors with consistent results *P< 0.01.

To determine the mechanism by which phospho-HCV E2 inhibited P+I induced IL-2 release, we assessed the activation and nuclear translocation of the nuclear factor of activated T cells (NFAT), a critical transcription factor required for IL-2 mRNA transcription. Upon P+I stimulation, NFAT was activated (dephosphorylated) similarly in control and HCV E2 protein expressing Jurkat cells (Fig 8A). However, nuclear translocation of active NFAT was reduced in HCV E2-expressing cells compared to that in control cells (Fig 8B). Since Y613 on E2 protein was phosphorylated by Lck and phospho-HCV E2 was required for reduced nuclear translocation of NFAT, interaction between NFAT and phosphorylated HCV E2 protein was assessed. HCV E2 protein did not precipitate NFAT in either unstimulated or TCR stimulated Jurkat cells (S9C Fig). NFAT nuclear import and export is regulated by large number of cellular proteins and non-coding RNAs, including importin-beta, tubulin-alpha, calcineurin, protein kinase D2 (PKD2), CSE1L, and others [45]. No direct interaction between either E2 or phospho-E2 with the factors studied to date was observed in immune precipitation experiments (S9C Fig). These data suggest that, upon phosphorylation at HCV Y613 by Lck, NFAT nuclear translocation is inhibited resulting in impaired distal TCR signaling.

Next, we assessed the effect of HCV enveloped particles on distal TCR signaling in primary human T cells. Following P+I stimulation, IL-2 release from healthy human PBMCs was inhibited by HCV particles obtained from serum (Fig 8C), and by infectious and defective HCV particles generated *in vitro* (HCVcc and HCVpp, respectively) (Fig 8D). Since mutation of Y613 to phenylalanine (Y613F) reversed the HCV E2-mediated inhibition of distal TCR signaling in Jurkat cells (Fig 7A), retroviral particles were pseudotyped with native E1-E2 or E1-E2 with the Y613F mutation (HCVpp Y613F). IL-2 release was restored in cells incubated with the Y613F mutant following P+I stimulation of healthy PBMCs (Fig 8D). Together, these data identify a single residue (Y613) on HCV envelope protein that is essential for inhibition of distal TCR signaling.

Taken together, these data confirm that HCV RNA-containing serum, HCVcc, HCVpp, HCV E2 protein, but not HCV E2 RNA inhibit distal TCR signaling in primary human T cells and the CD4+ human T cell line. This inhibition requires Lck phosphorylation of Y613 of HCV E2 protein, and does not require viral replication, as HCVpp inhibit distal signaling.

## Discussion

HCV establishes persistent infection through complex and incompletely understood mechanisms. A strong T cell response correlates with effective control and clearance of HCV infection; however, most infected individuals fail to clear viremia [2]. Chronic infection is associated with a reduction in HCV-specific intrahepatic and peripheral blood T cell functions, suggesting that HCV proteins, RNA, or both inhibit normal T cell function. Here we demonstrate that HCV particles directly reduced T cell activation via inhibition of the T cell receptor (TCR) signaling pathway. Serum, HCV RNA-containing EV from HCV-infected individuals and HCVcc inhibited TCR signaling in human T cells. Inhibition of TCR signaling did not require replication, as replication incompetent HCVpp inhibited TCR signaling. The relative inhibition of TCR signaling by HCV positive sera was stronger than that mediated by cell culture derived HCVcc or HCVpp. This is not surprising, since HCV positive sera contain TCR inhibitory cytokines (IL-10, TGF-beta) that are not present in HCVcc, purified EVs and HCVpp [46,47]. Nevertheless, in the absence of these serum factors, HCVccs, HCV positive EVs and HCVpps inhibited TCR signaling in PBMCs and in purified T cells, thus viral E2 protein and RNA were sufficient to alter TCR signaling.

HCV RNA encoding E2 and E2 protein itself independently inhibited TCR signaling at two distinct sites within the TCR signaling pathway. HCV E2 RNA inhibited proximal TCR signaling by reducing activation of Lck, and inhibition required highly conserved nucleotide sequences flanking the conserved E2 Y613. The RNA region contains conserved residues predicted form a structure processed by Dicer into a vd-sRNA. Mutation of four of the conserved residues abolished the predicted RNA secondary structure and restored TCR signaling. Conserved sequences in the vd-sRNA sequence were predicted to target PTPRE, a phosphatase involved in Src kinase signaling.

PTPRE translation was significantly reduced in lymphocytes expressing HCV E2 RNA and in hepatocytes expressing the HCV full-length genome. PTPRE regulates Src signaling through Grb2 [44], and Grb2 deficient cells have impaired Lck activation [48], suggesting that HCV-derived, short RNA interferes with PTPRE translation leading to reduced TCR signaling. HCV replication in hepatocytes is enhanced by inhibition of Src kinases [49], thus knockdown of hepatocyte PTPRE which regulates Src kinases by this HCV-derived short RNA may also facilitate viral replication in hepatocytes in addition to interfering with T cell signaling in lymphocytes. The addition of HCV RNA-containing serum to 293 cells targeted PTPRE sequences when added to GFP compared to HCV RNA negative serum, illustrating the biological relevance of this observation.

A second mechanism of inhibition of TCR signaling involved E2 protein phosphorylation. Four lines of evidence in these studies support a role for Lck-mediated phosphorylation of HCV E2 protein at Y613. First, E2 was phosphorylated *in vitro* by Lck. Second, native E2 protein expressed in Jurkat cells was phosphorylated following TCR engagement yet E2 with a Y613A mutation was not. Third, an Lck inhibitor rescued the HCV E2 protein-mediated effect on NFAT translocation following P+I activation, and finally, mutation of Y613 to alanine or phenylalanine restored distal TCR signaling. Phosphorylated HCV E2 inhibited distal TCR signaling by reducing NFAT nuclear translocation. NFAT nuclear import and export is regulated by large number of cellular proteins and non-coding RNAs [45]. Phospho-E2 may interfere with any of these factors, or combinations of factors, resulting in impaired NFAT nuclear translocation. Current studies are underway to characterize this further.

**Fig 9:**
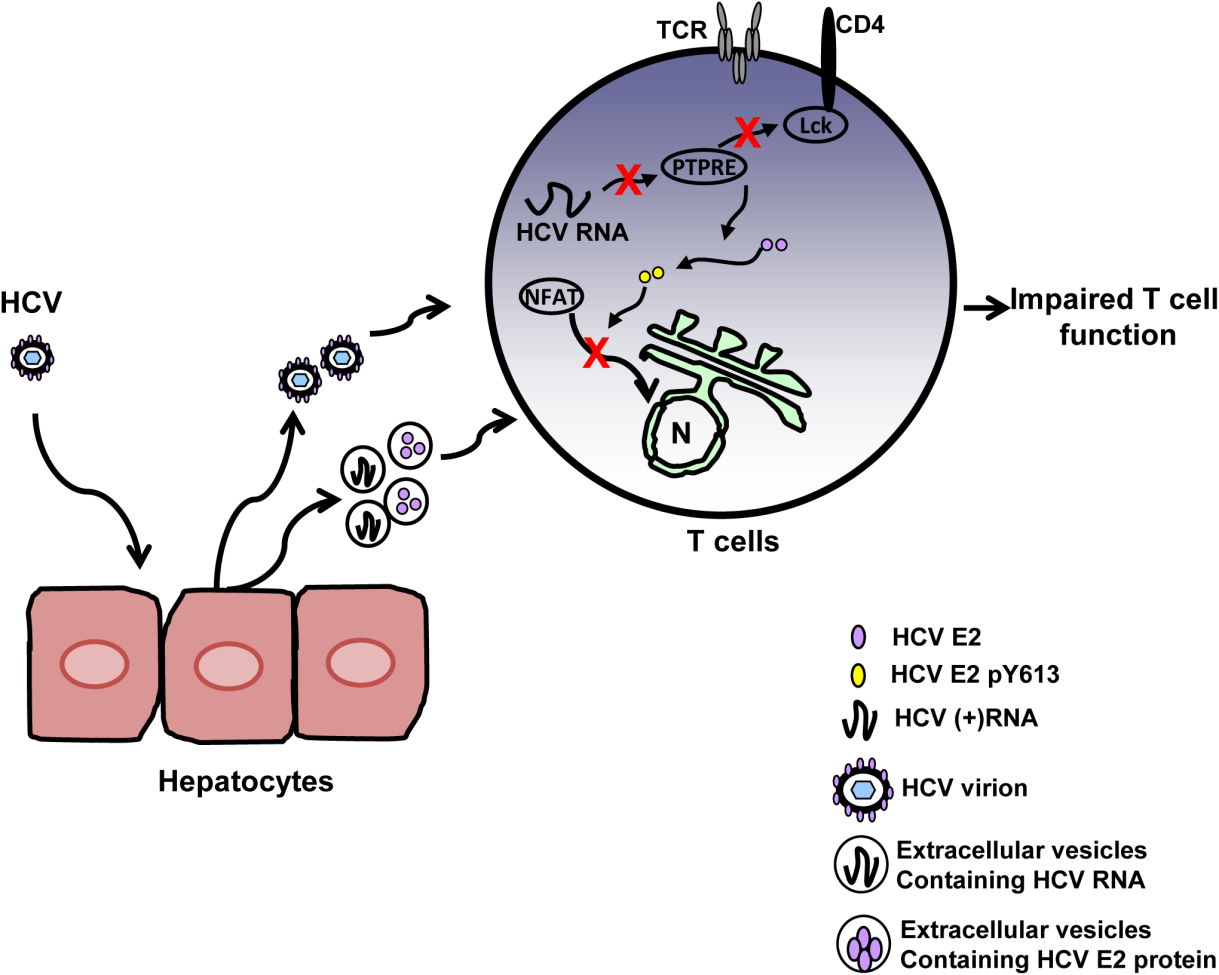
Proposed model for inhibition of T cell receptor (TCR) signaling during HCV infection. HCV infection of hepatocytes results in the release of progeny HCV virions and extracellular vesicles containing HCV RNA and/or E2 protein. Viral RNA and/or E2 protein is released into T cells during particle interactions. HCV envelope RNA is processed into small RNA that inhibits protein tyrosine phosphatase E (PTPRE) expression, which results into impaired Lck activation following TCR engagement and defect in proximal TCR signaling. HCV E2 protein competes for Lck-mediated phosphorylation and phosphorylated HCV E2 at Y613 inhibits NFAT nuclear translocation, inhibiting distal TCR signaling. Inhibition of proximal and distal TCR signaling by HCV E2 RNA and protein contributes to impaired T cell function during HCV infection.

Together, the results indicate that HCV E2 Y613 served as an Lck substrate, and that Lck-mediated phosphorylation of Y613 was required for distal TCR signaling inhibition. Although, the amount of viral RNA and E2 protein transferred to the target T cells is unlikely to be comparable to that present in Jurkat cells expressing E2, significant inhibition of T cell activation was measured in cells incubated with serum, EVs or viral particles (HCVccs and HCVpps) following stimulation with potent TCR agonists (CD3/CD28 antibodies). Thus the small amount of E2 protein and RNA present in serum and EVs is sufficient to reduce TCR activation. Although E1 could influence T cell activation inhibition, E2 protein was sufficient to inhibit distal TCR signaling, and mutation of the Y613 residue restored TCR signaling in the context of E2 protein expression (Y613A, Y613F) or HCV particles (HCVpp; Y613F). A model illustrating HCV particle interactions and the steps of E2 RNA and protein inhibition of TCR signaling is shown in Fig 9. Finally, the fact that HCV E2 RNA and protein inhibited TCR-mediated activation at two distinct steps in the signaling cascade highlights a critical role for TCR in HCV infection. The RNA and amino acid inhibitory sequences are highly conserved, suggesting that both mechanisms of TCR inhibition are synergistic in cells expressing HCV E2 RNA and protein.

During HCV infection, the concentration of serum HCV RNA-containing particles is high, frequently greater than one million copies of viral RNA per milliliter [50,51]. Thus there are abundant interactions between viral RNA-containing particles and lymphocytes. In addition to virions, HCV RNA is also present in extracellular vesicles [15,16,19]. Extracellular vesicles containing HCV, HPgV or hepatitis A virus RNA have been shown to deliver viral RNA into uninfected cells and initiate infection [15,16,52,53]. Although, *in vitro* effects of HCV sera on TCR appears to be potent and dose-dependent (Fig 1C), the inhibitory effects of HCV RNA and protein are not complete *in vivo* because the concentration of HCV RNA and protein in lymphocytes is low. Thus, the inhibitory effect of HCV particles does not lead to severe immune deficiency.

Nevertheless, there is evidence of mild immune suppression during HCV infection. Specifically, HCV infected subjects have blunted immune responses to vaccine antigens like HBV and reduced organ transplant rejection [1,12–14]. The reduction in T cell activation and IL-2 release mediated by HCV particles may also contribute to impaired T cell proliferation, differentiation and effector function observed in prior studies [54,55], which may aid in the establishment of acute HCV infection and contribute to viral persistence during chronic infection. Furthermore, efficient T cell activation is required for the generation of an effective immune response against pathogens, including vaccine antigens. Thus, mutation of TCR inhibitory motifs within HCV E2 RNA and protein may potentially lead to the design of improved envelope-based HCV vaccines.

## Materials and Methods

### Cells and viruses

Human hepatocellular carcinoma cell line (Huh-7.5; kindly provided by Dr. Charles Rice, The Rockefeller University) was cultured in Dulbecco's modified Eagle's medium (DMEM) containing 10% fetal bovine serum, 1% penicillin-streptomycin and 1% L-glutamine at 37°C in a 5% CO_2_. HCV positive human serum infected with genotypes (1, 1a, 1b, 2, 2b and 3) or negative control serum was prepared from blood obtained from patients or from healthy blood donors. Huh 7 cells containing replicons consisting of either the full length HCV genome or the NS2-5 region of the genomes were kindly provided by Dr. Ralf Bartenschlager (University of Heidelberg) and maintained as described [56,57]. Serum extracellular vesicles (EV) were purified from serum using the ExoQuick reagent (Systems Biosciences) according to the manufacturer’s instructions. Specifically, human sera was incubated with the ExoQuick reagent for 1 hr. (4°C) and centrifuged 30 minutes (10,000g) as recommended. The pellet was resuspended in RPMI and stored at -20°C until use. This reagent has been reported to yield EVs from both cell culture supernatant and human serum [32]. Cell culture derived, infectious HCV particles (HCVcc) were obtained by transfecting Huh7.5 cells with *in vitro* transcribed HCV RNA from J6/JFH infectious clone (kindly provided by Dr. Takaji Wakita, Tokyo Metropolitan Institute of Neuroscience, and Dr. Charles Rice, Rockefeller University) as described by others [58]. Cell culture supernatant was harvested 72 hours following transfection and concentrated. The HCV titer in the culture supernatant was 4.98×10^7^ (copies/mL). 4.98×10^7^ particles were added to 1×10^6^ cells. HCV (E1-E2) pseudotyped HIV particles (HCVpp) or HIV gag particles without a viral envelope (GAGpp) were generated in HEK 293T cells using pNL4-3-Luc.R-E- (NIRRRP catalog # 3417) as described [59]. HCVcc, HCVpp and GAGpp in supernatants were concentrated using Amicon 100K filter units (Millipore) and HCVpp/ GAGpp were quantified using p24 ELISA (Zeptometrix Inc.).

### Expression of HCV envelope protein

Coding regions of HCV E2 protein from J6/JFH plasmid (aa 384–747) [58] or from a genotype 3 isolate obtained from a patient from the University of Iowa were amplified and ligated into a modified pTRE2-HGY plasmid (Clontech, Inc.) as previously described [60]. HCV sequences were confirmed by sequencing plasmid DNA (University of Iowa DNA Core Facility). The modified plasmid generates a bicistronic message encoding the HCV E2 sequence followed by stop codons, the encephalomyocarditis virus (EMC) internal ribosomal entry site (IRES) directing the translation of GFP. Jurkat (tet-off) cell lines (Clontech, Inc) were transfected (Nucleofector II, Lonza Inc.) and cell lines were selected for hygromycin and G418 resistance. GFP positive cells were bulk sorted (BD FACS Aria, (University of Iowa Flow Cytometry Facility) and GFP expression was assessed by flow cytometry (BD LSR II). HCV E2 protein expression was determined by immunoblot using human monoclonal antibodies (HC33-1, kindly provided by Dr. Steven Foung, Stanford University). All cell lines were maintained in RPMI 1640 supplemented with 10% heat-inactivated fetal calf serum, 2mM L-glutamine, 100 IU/ml penicillin, and 100 μg/ml streptomycin with hygromycin and G418 (200 μg/ml).

### Cell isolation and stimulation

Peripheral blood mononuclear cells (PBMCs) were prepared from blood obtained from healthy donors by Ficoll-gradient centrifugation. PBMCs were incubated with HCV positive or negative serum (100μl for each unless otherwise stated) overnight. CD3^+^ (T) cells were enriched by positive selection using magnetic system according to manufacturer’s instructions (Miltenyi Biotec, Auburn, CA), and purity assessed by flow cytometry. PBMCs (1×10^6^ cells/ml) were stimulated with plate-bound anti-CD3 (100ng/ml, OKT3 clone, eBioscience) and soluble CD28 antibody (100ng/ml, clone CD28.2, BD Biosciences). Jurkat cells (5×10^6^ cells/ml) were stimulated with anti-CD3 and soluble CD28 (both at 5μg/ml) or phorbol-12-myristate-13-acetate (PMA, 50ng/ml) and ionomycin (1μg/ml) (P+I). Cellular receptor expression and cytokine release were measured 24 hours post-stimulation by flow cytometry and ELISA respectively. For Lck inhibition, Jurkat cells were incubated with Lck inhibitor II (EMD Millipore) at 100μg/ml overnight before stimulating with P+I.

### Flow cytometry

Cell surface receptor expression was measured with CD69 (APC), or CD45 (PE) antibodies (BD Biosciences) using the manufacturer’s recommendations. Cells were incubated on ice for 1 hour, washed 3 times with PBS and fixed in 2% paraformaldehyde (Polysciences). Purified extracellular cellular vesicles (EV) were stained with either anti-CD63 exo-flow staining kit (Systems Biosciences) or CFSE dye (5μM) for 15 minutes at 37°C. EVs were washed in PBS four times and concentrated using Amicon 100K filter units (Millipore). Data was acquired on BD LSR II flow cytometer using single stained CompBeads (BD Biosciences) for compensation. At least 10,000 total events were collected in each experiment and the FlowJo software program (Tree Star Inc.) was used for data analysis. All flow cytometry experiments were repeated at least three times with consistent results.

### HCV PCR

After overnight incubation, PBMCs were incubated in trypsin for 1 minute and washed twice with RPMI. Total RNA was isolated (RNeasy Kit, Qiagen) and cDNA was made with HCV 5’ UTR specific primers or random hexamers. For first round RT-PCR, the outer primers were sense 5‘CTCCACCCAATGAATCACTCCC and antisense 5’GAGGTTTAGGATTCGTGCTC. For nested PCR, the primers were sense 5’CGTTAGTATGAGTGTCGTGC and antisense 5’GATGCACGGTCTACGAGACC. The final product size was 250bp. GAPDH primers used were sense 5’ATCCCATCACCATCTTCCAG and antisense 5’CCATCACGCCACAGTTTCC which generates a product size of 383bp.

HCV E2 derived small RNAs were identified as follows, total RNA from Jurkat cells expressing HCV E2 was isolated (RNeasy Kit, Qiagen). RNA was ligated to a pre-adenylated DNA universal miRNA cloning linker (New England Biolabs) using T4 RNA ligase 2 (New England Biolabs) according to the manufacture’s protocol. Ligated RNA was purified using RNA columns (Qiagen) and cDNA transcribed using a DNA linker primer (5’- ATTGATGGTGCCTACAG-3’). PCR was carried out using HCV E2 primer (5’-TCCTGATACCACTTACCTCAA-3’) and DNA linker primer. PCR products were cloned into TA cloning vector (Invitrogen) and DNA sequences were obtained by sequencing plasmid (University of Iowa DNA Core Facility).

### ELISA and immunoblot analyses

IL-2 cytokine released into cell culture supernatant was quantified using human IL-2 ELISA kit (BD Biosciences) according to the manufacturer’s instructions. Jurkat cells were stimulated with anti-CD3 (5 μg/ml) for the indicated times prior to addition of cell lysis buffer (Cell Signaling). Following PMA/Ionomycin stimulation for 15 minutes, nuclear proteins were isolated using nuclear protein isolation kit (NEPER, Thermo Scientific) following manufacturer’s instructions. Proteins were separated by polyacrylamide gel electrophoresis and transferred to nitrocellulose membranes (BIORAD). Membranes were incubated in protein-free blocking buffer (Thermo Scientific) for 1 hour at room temperature followed by incubation with primary antibodies. Proteins were detected with Amersham ECL (GE Healthcare) using a Kodak Imager. Primary antibodies used were: NFAT and pLAT(Y226; BD Biosciences); total LAT (Biolegend); pZAP70 (Y319); total ZAP70; pLck (Y394/ pSrcY416); total Lck (Y394); Csk; YY1 (all from Cell Signaling Technology); PTPRE (Origene, clone 4B2). Immunoblots were quantified by ImageJ.

### *In vitro* kinase assay

Phosphorylation of HCV E2 protein by Lck was measured by incubating recombinant E2 protein (eEnzyme) with or without human Lck (R&D Systems) and CD45 (Enzo Life Sciences) as recommended by the manufacturer. Samples were subjected to immunoblot analysis as described above. Phosphorylation was determined by immunoblot analysis with phosphotyrosine antibodies (Invitrogen) and HCV E2 protein was identified using anti-HCV E2 human monoclonal antibodies described above.

### Immunoprecipitation

Jurkat cells stably expressing HCV E2 (aa 384–715 of the HCV polyprotein) with a C-terminal influenza hemagglutinin (HA) tag were stimulated with 10μg/ml of anti-CD3 for 15 minutes and lysed (25mM Tris, 150mM NaCl, 5% Glycerol, 1mM EDTA, 1% NP40; 1 hour on ice). Cell lysates were incubated with anti-HA agarose beads (Thermo Scientific) or NFAT antibodies conjugated to protein G beads at 4°C overnight. Beads were pelleted and washed 3 times in lysis buffer. Bound proteins were eluted in 2x Laemmle sample buffer prior to immunoblot analysis.

### Statistics

All data represent the average of three technical replicates unless otherwise stated, and error bars are provided to show the standard deviation. Each experiment was independently performed at least three times with consistent results. For experiments utilizing primary human PBMCs, at least three different healthy blood donors were studied. Statistics were performed using GraphPad software V4.0 (GraphPad Software Inc.). Two-sided Student’s t test was used to compare results between test and controls. *P* values less than 0.05 were considered statistically significant.

### Ethics statement

This study was approved by the University of Iowa Institutional Review Board. All subjects (healthy donors and subjects with viral infections) provided written informed consent.

## Supporting Information

S1 Fig Purity of CD3+ T lymphocytes following purification. Representative flow cytometry analysis of CD3 staining in T cells obtained from healthy blood donor before and after purification.
(PDF)

S2 Fig Characterization of extracellular vesicles (EVs) purified from serum. Flow cytometry analysis of CD63, CD81, CD69 and CD25 expression in EVs obtained from HCV positive human serum (A). HCV RNA quantification in PBMCs and purified CD3+ T cells obtained from two subjects with and without HCV infection (B). HCV RNA was amplified from RNA extracted from PBMCs of 6 HCV infected individuals (C).
(PDF)

S3 Fig HCV particles inhibit T cell receptor signaling in purified human T cells. HCV cell culture-derived infectious particles (HCVcc, panel A) and HCV envelope pseudotyped retrovirus particles (HCVpp, panel B) inhibited IL-2 release following stimulation with anti-CD3/CD28 in purified human CD3+ T cells. Data represent the average of three technical replicates and the standard deviation is shown. Each experiment was independently performed with two different donors. *P< 0.05.
(PDF)

S4 Fig Expression of HCV and effect of E2 on the activation of TCR signaling molecules. Immunoblot analysis of HCV E2 and GAPDH expression in Jurkat cells expressing HCV E2 or Jurkat control cells (JC) (A). CD69 surface expression after 24 hours of CD3/CD28 stimulation of Jurkat cells (B). Schematic diagram illustrating the regions of HCV E2 protein expressed in the Jurkat cell lines generated (C).
(PDF)

S5 Fig Expression of GFP and HCV E2 proteins in Jurkat cells. GFP expression in Jurkat cell lines stably transfected with plasmid encoding various HCV E2 fragments as determined by flow cytometry (A). Schematic diagram illustrating the tyrosine 613 mutations expressed in the Jurkat cell lines (B). Immunoblot analysis of Jurkat cell lines stably transfected with plasmid encoding GFP (JC), HCV E2 protein (HCV E2), HCV E2 RNA in which a frame-shift mutation was inserted (HCV E2 RNA), or mutant E2 expressing Y613A or Y613F (C).
(PDF)

S6 Fig Effect of HCV E2 protein on Lck regulatory proteins. Expression of CD45 as determined by flow cytometry in HCV E2 and JC cells (A). C-terminal Src kinase (Csk) expression measured by immunoblot analysis in HCV E2 and JC cells (B).
(PDF)

S7 Fig Predicted structure and Dicer cleavage sites for HCV E2 RNA motif that inhibits proximal TCR signaling. The predicted HCV RNA structure of sequences encoding amino acids 603–619 for genotype (GT) 2a, 3, and the GT 2a mutant are shown. The arrow identifies the predicted cleavage site of Dicer, and the (X) indicates that the predicted Dicer cleavage site is abolished in the mutant. * = Mutations introduced into HCV E2 RNA.
(PDF)

S8 Fig PTPRE mRNA is not altered by HCV E2 RNA. Steady-state mRNA levels of protein tyrosine phosphatase (PTPRE) in Jurkat cells expressing HCV E2 native or mutant RNA and controls. PTPRE expression was normalized to actin.
(PDF)

S9 Fig HCV E2 protein, signaling to CD69, and interactions with NFAT regulatory molecules. Jurkat cell lines expressing HCV E2 (384–747) or the E2 region coding RNA with a frameshift mutation to abolish protein expression (E2 RNA) or HCV E2 with a phenylalanine substitution for Y613 (384–703 Y613F) did not inhibit CD69 expression in Jurkat cells following PMA and Ionomycin (P+I) stimulation (A). NFAT was precipitated by anti-NFAT antibody as described in methods. HCV E2 and NFAT precipitation was analyzed by immune blot. Interactions between HCV E2 and NFAT were not detected in Jurkat cells expressing HCV E2 (384–703) or the mutant HCV E2 (Y613F) with or without CD3 stimulation by co-immune precipitation (B). NFAT and HCV present in the original cell lysate (lysate) and in lysates incubated with non-specific control antibody (IgG) are shown. Immunoblot analysis of HA-tagged HCV E2 protein with cellular proteins that regulate NFAT nuclear translocation following CD3 stimulation (C).
(PDF)

S10 Fig HCV E2 protein inhibits proximal, but not distal activation of CD69. Representative plots of CD69 surface expression on Jurkat cell lines expressing HCV E2 (384–747) or the Jurkat control cells expressing only GFP (JC) before stimulation and after stimulation with anti-CD3/CD28 or PMA/Ionomycin for 24 hours. Each experiment was repeated at least three times with consistent results.
(PDF)
