# Supplementary figures and images for: Conserved Motifs within Hepatitis C Virus Envelope (E2) RNA and Protein Independently Inhibit T Cell Activation
Source: PLoS Pathog. 2015 Sep 30;11(9):e1005183. doi: 10.1371/journal.ppat.1005183 (PMC4589396; doi:10.1371/journal.ppat.1005183)

**A.**

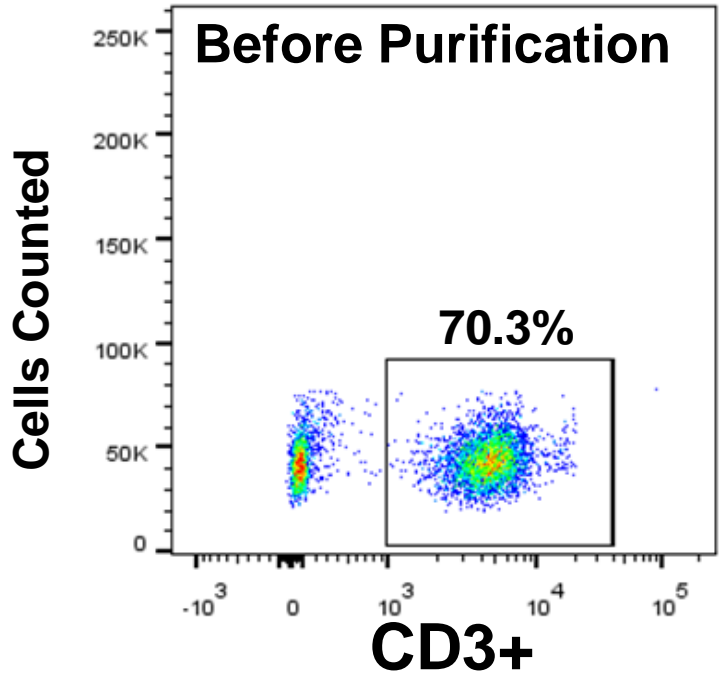

**B.**

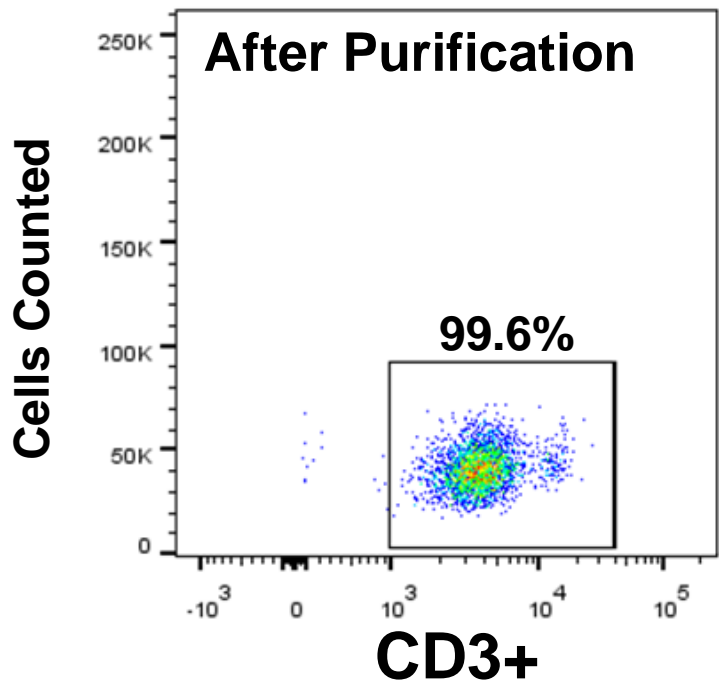

Supplement: S1 Fig — Representative flow cytometry analysis of CD3 staining in T cells obtained from healthy blood donor before and after purification. (PDF) [file ppat.1005183.s001.pdf]

**A.**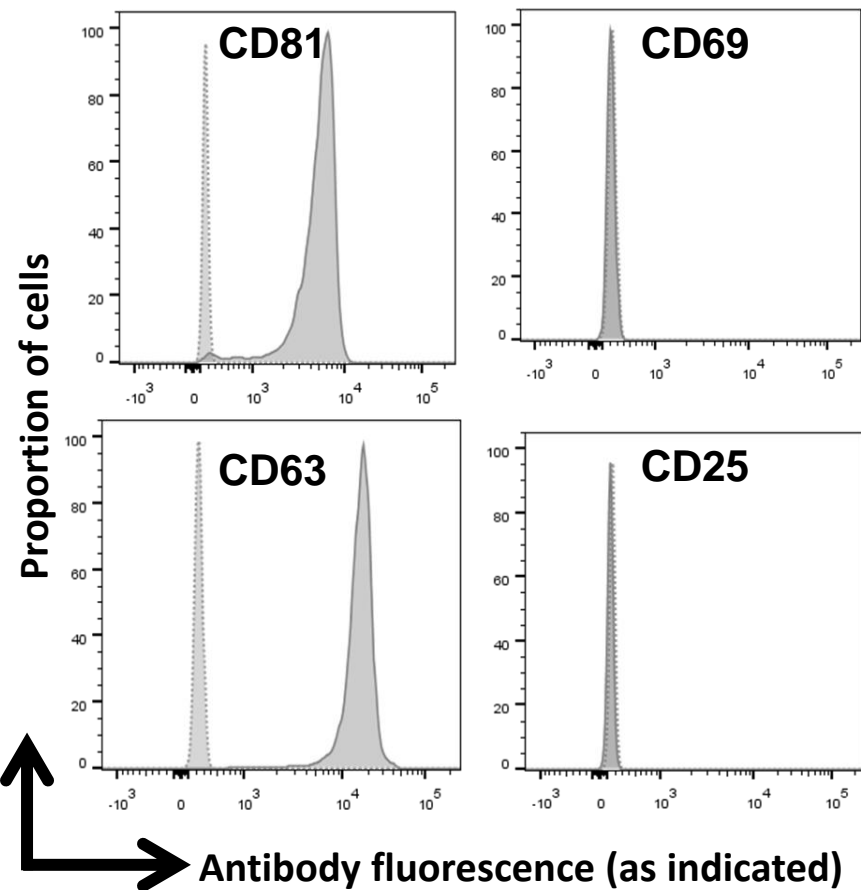**B.**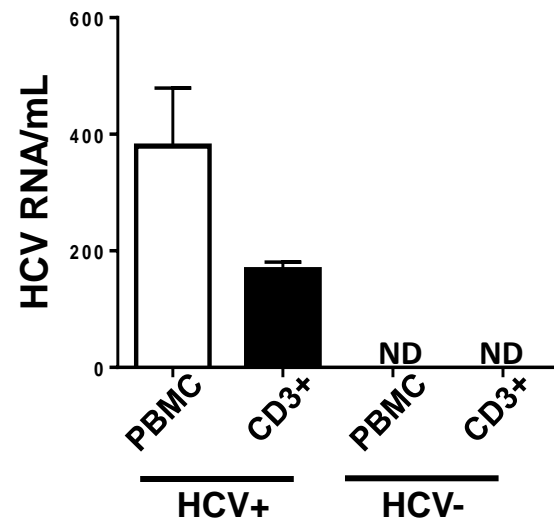**C.**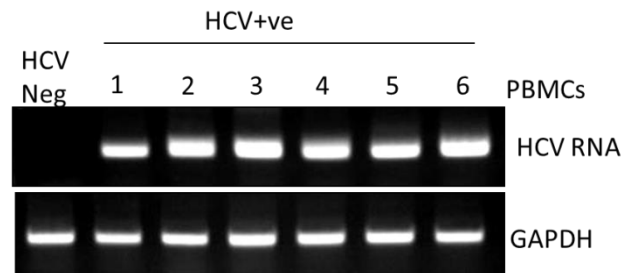

Supplement: S2 Fig — Flow cytometry analysis of CD63, CD81, CD69 and CD25 expression in EVs obtained from HCV positive human serum (A). HCV RNA quantification in PBMCs and purified CD3+ T cells obtained from two subjects with and without HCV infection (B). HCV RNA was amplified from RNA extracted from PBMCs of 6 HCV infected individuals (C). (PDF) [file ppat.1005183.s002.pdf]

**A.****CD3+ T cells**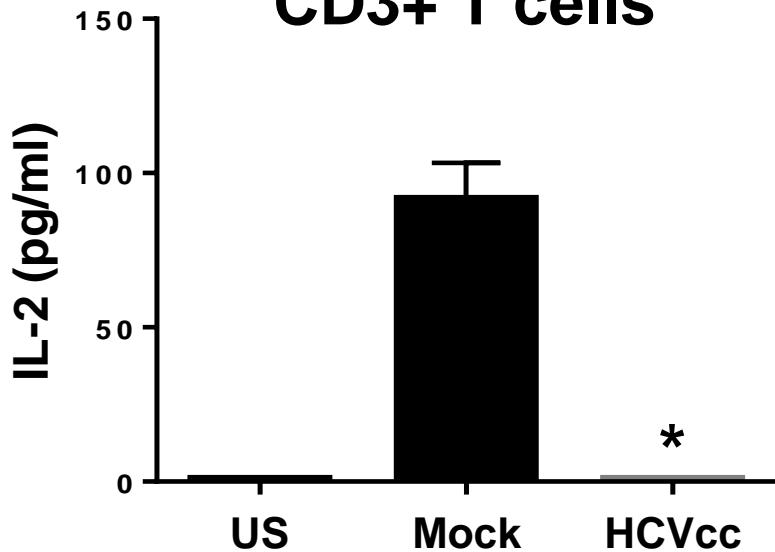**B.****CD3+ T cells**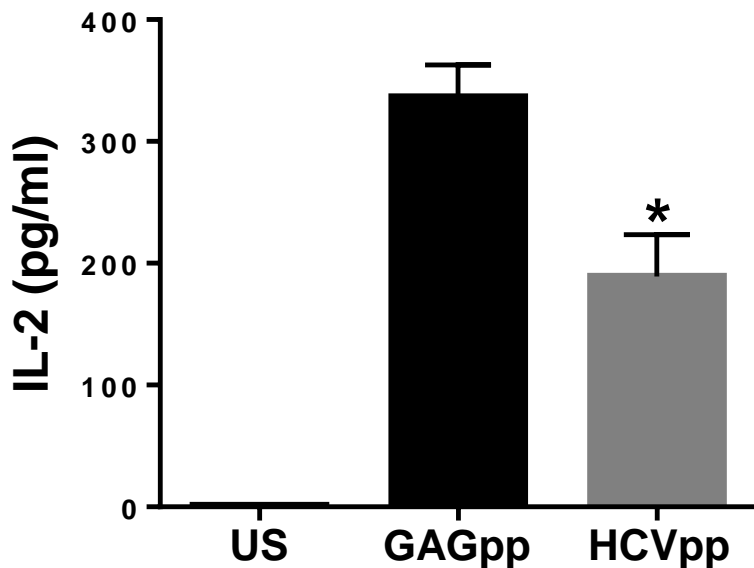

Supplement: S3 Fig — HCV cell culture-derived infectious particles (HCVcc, panel A) and HCV envelope pseudotyped retrovirus particles (HCVpp, panel B) inhibited IL-2 release following stimulation with anti-CD3/CD28 in purified human CD3+ T cells. Data represent the average of three technical replicates and the standard deviation is shown. Each experiment was independently performed with two different donors. *P< 0.05. (PDF) [file ppat.1005183.s003.pdf]

**A.**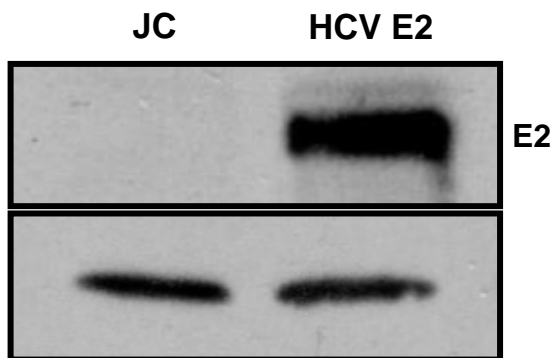**B.**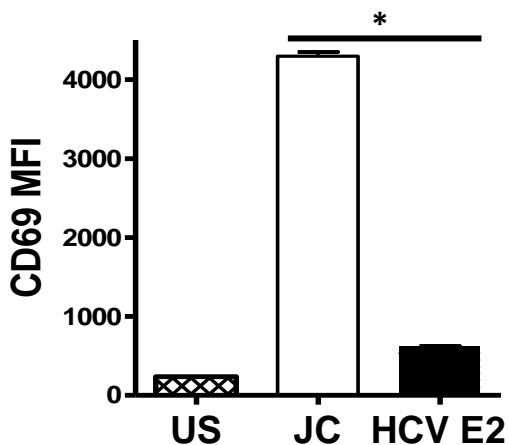**C.**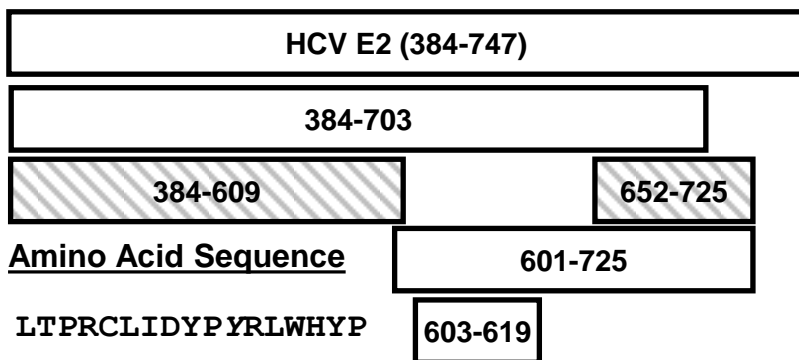

Supplement: S4 Fig — Immunoblot analysis of HCV E2 and GAPDH expression in Jurkat cells expressing HCV E2 or Jurkat control cells (JC) (A). CD69 surface expression after 24 hours of CD3/CD28 stimulation of Jurkat cells (B). Schematic diagram illustrating the regions of HCV E2 protein expressed in the Jurkat cell lines generated (C). (PDF) [file ppat.1005183.s004.pdf]

**A.**

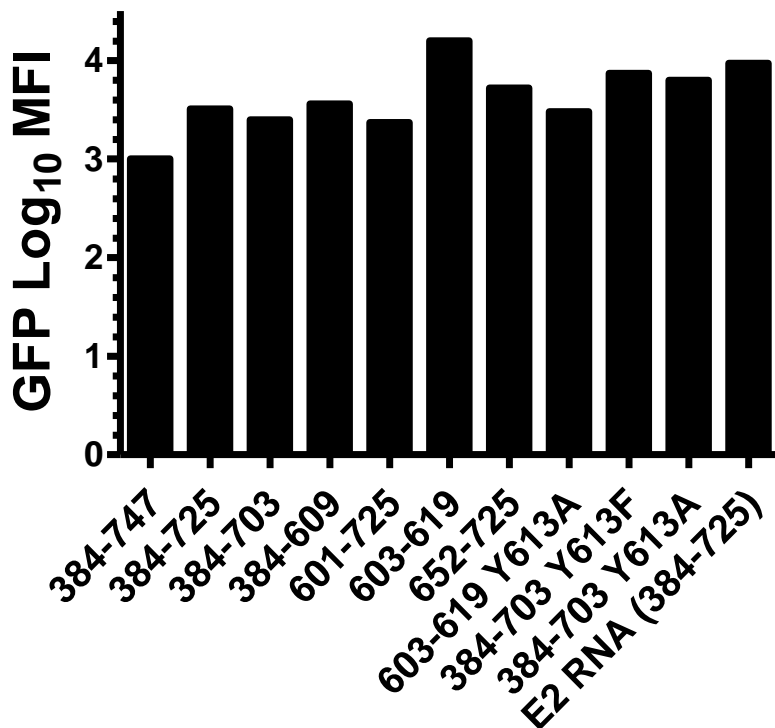

**B.**

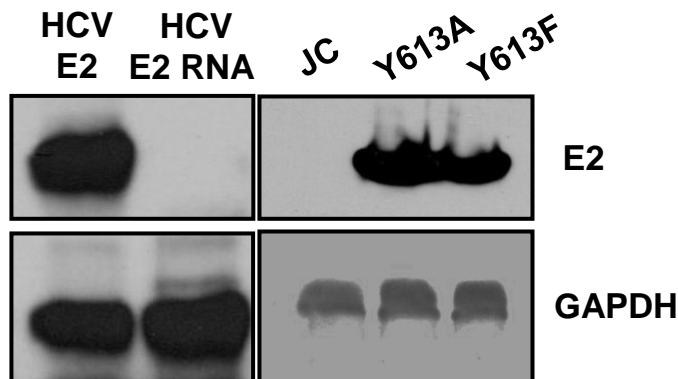

Supplement: S5 Fig — GFP expression in Jurkat cell lines stably transfected with plasmid encoding various HCV E2 fragments as determined by flow cytometry (A). Schematic diagram illustrating the tyrosine 613 mutations expressed in the Jurkat cell lines (B). Immunoblot analysis of Jurkat cell lines stably transfected with plasmid encoding GFP (JC), HCV E2 protein (HCV E2), HCV E2 RNA in which a frame-shift mutation was inserted (HCV E2 RNA), or mutant E2 expressing Y613A or Y613F (C). (PDF) [file ppat.1005183.s005.pdf]

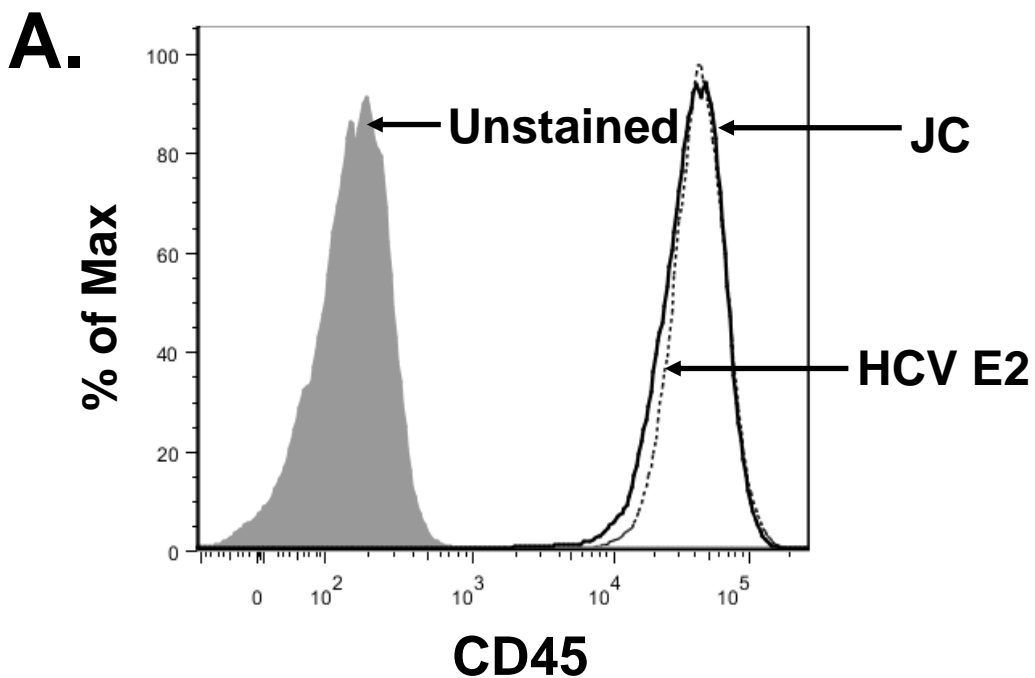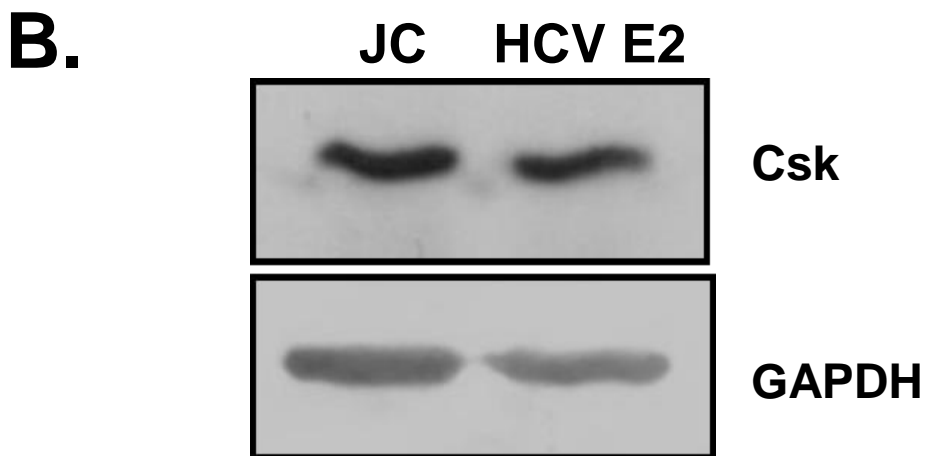

Supplement: S6 Fig — Expression of CD45 as determined by flow cytometry in HCV E2 and JC cells (A). C-terminal Src kinase (Csk) expression measured by immunoblot analysis in HCV E2 and JC cells (B). (PDF) [file ppat.1005183.s006.pdf]

**GT-2a**

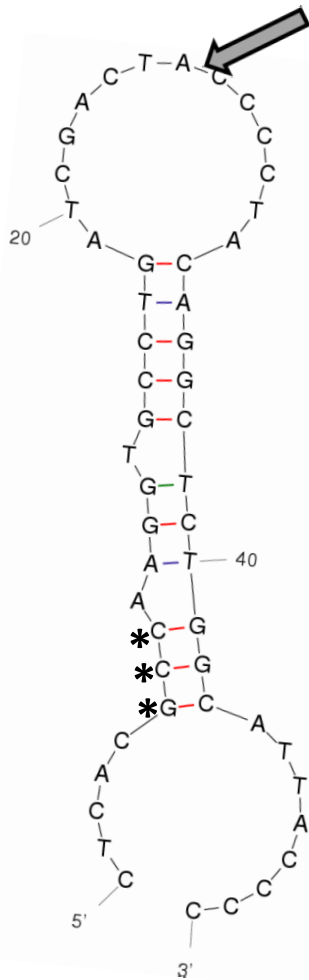

**GT-3**

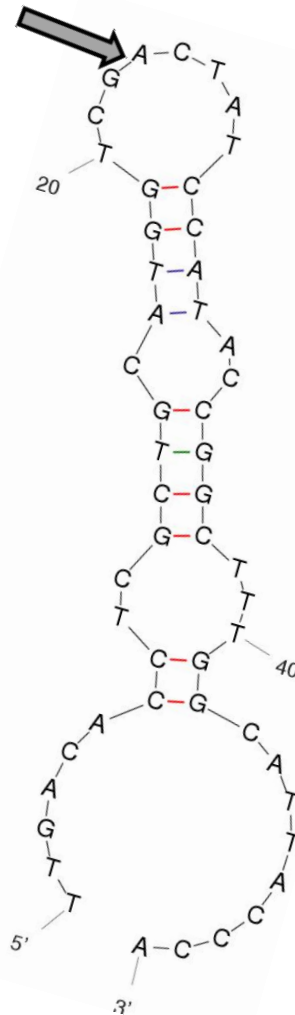

**GT-2a:  
Mutant**

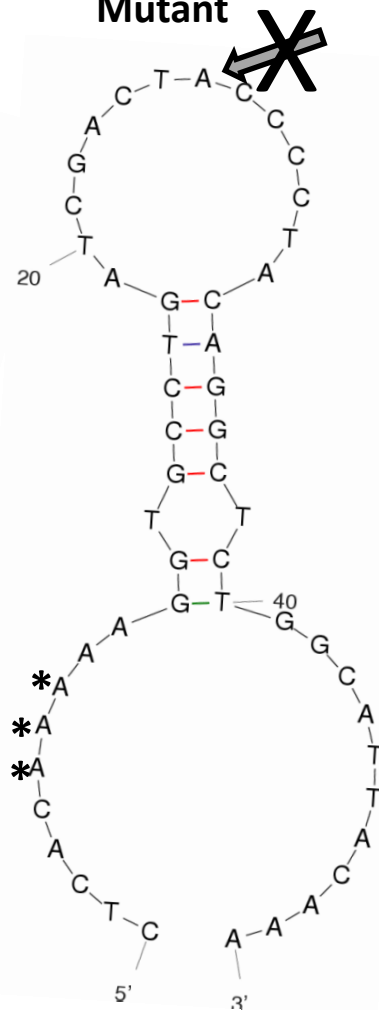

Supplement: S7 Fig — The predicted HCV RNA structure of sequences encoding amino acids 603–619 for genotype (GT) 2a, 3, and the GT 2a mutant are shown. The arrow identifies the predicted cleavage site of Dicer, and the (X) indicates that the predicted Dicer cleavage site is abolished in the mutant. * = Mutations introduced into HCV E2 RNA. (PDF) [file ppat.1005183.s007.pdf]

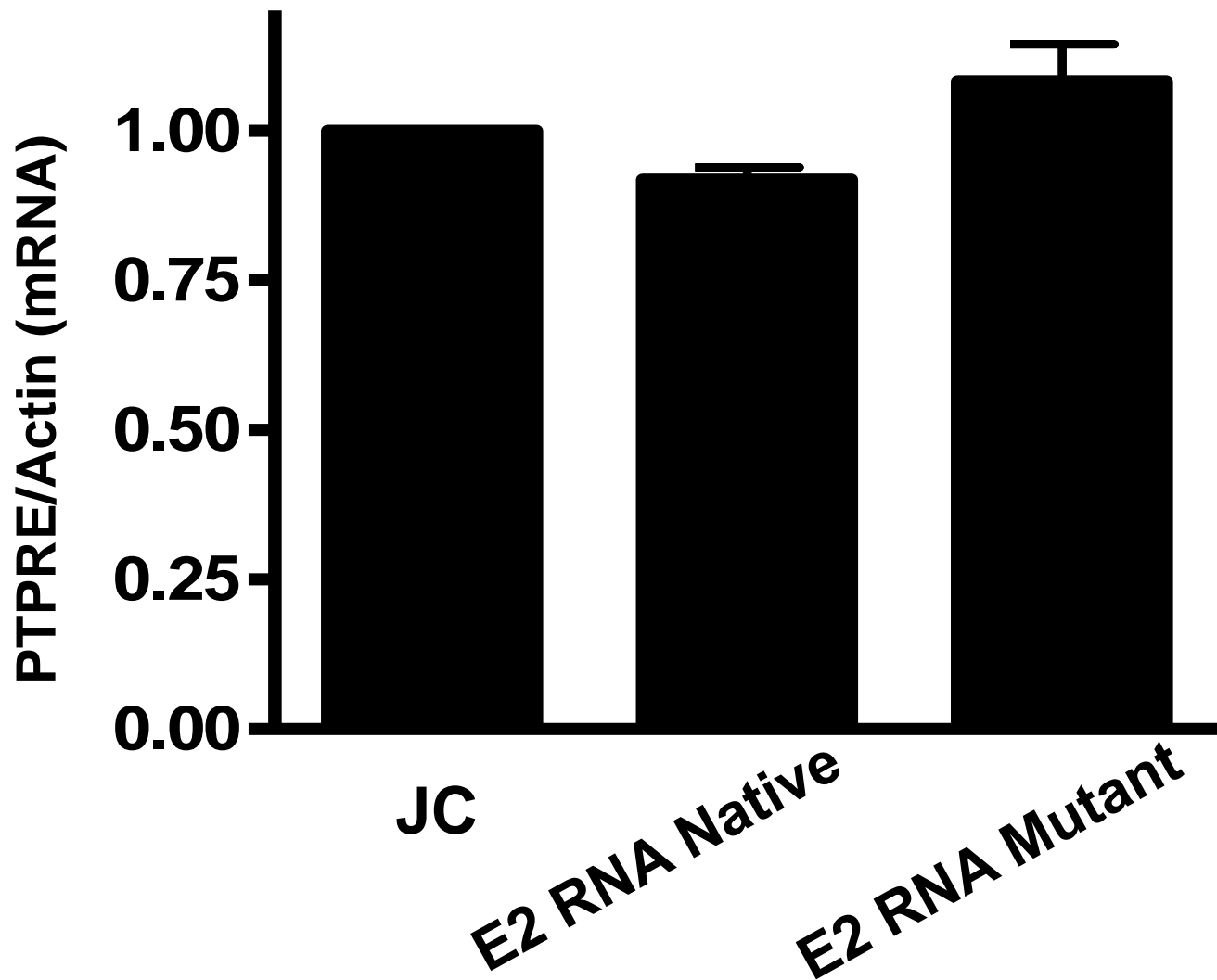

Supplement: S8 Fig — Steady-state mRNA levels of protein tyrosine phosphatase (PTPRE) in Jurkat cells expressing HCV E2 native or mutant RNA and controls. PTPRE expression was normalized to actin. (PDF) [file ppat.1005183.s008.pdf]

**A.**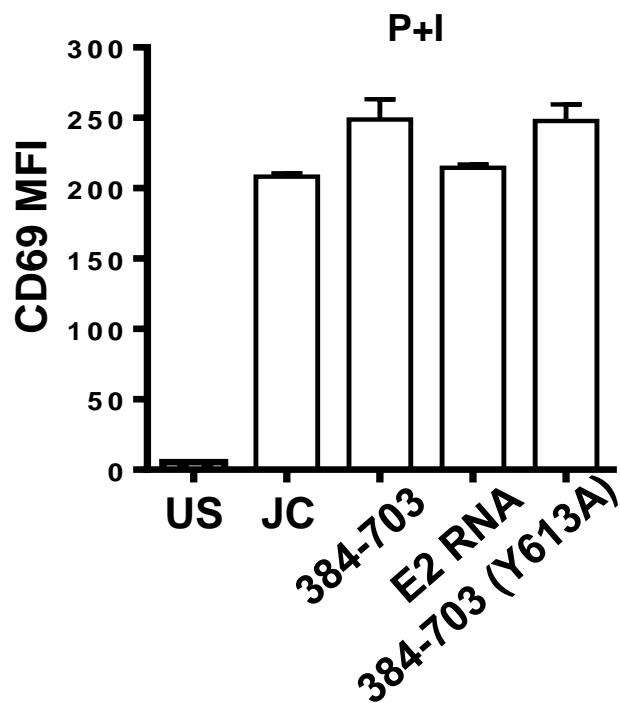**B.**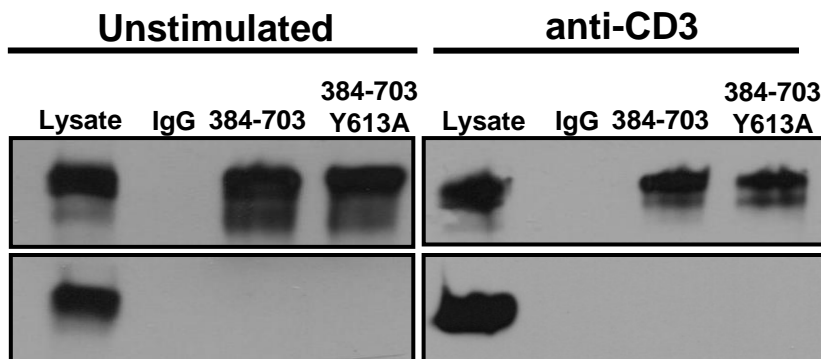**C.**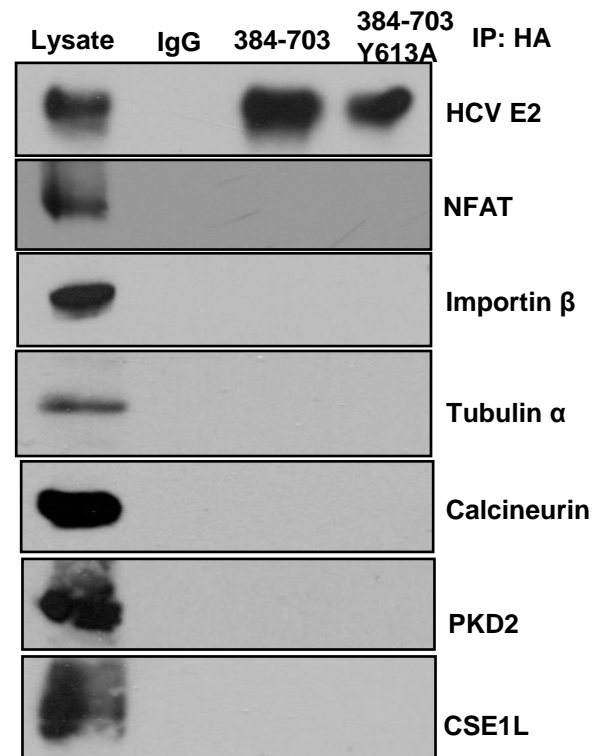**IP: NFAT**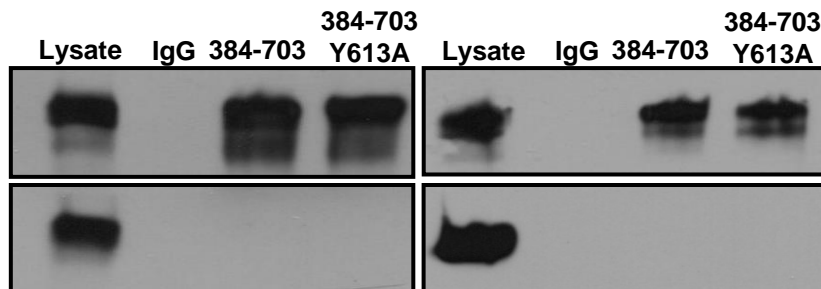**NFAT****HCV E2**

Supplement: S9 Fig — Jurkat cell lines expressing HCV E2 (384–747) or the E2 region coding RNA with a frameshift mutation to abolish protein expression (E2 RNA) or HCV E2 with a phenylalanine substitution for Y613 (384–703 Y613F) did not inhibit CD69 expression in Jurkat cells following PMA and Ionomycin (P+I) stimulation (A). NFAT was precipitated by anti-NFAT antibody as described in methods. HCV E2 and NFAT precipitation was analyzed by immune blot. Interactions between HCV E2 and NFAT were not detected in Jurkat cells expressing HCV E2 (384–703) or the mutant HCV E2 (Y613F) with or without CD3 stimulation by co-immune precipitation (B). NFAT and HCV present in the original cell lysate (lysate) and in lysates incubated with non-specific control antibody (IgG) are shown. Immunoblot analysis of HA-tagged HCV E2 protein with cellular proteins that regulate NFAT nuclear translocation following CD3 stimulation (C). (PDF) [file ppat.1005183.s009.pdf]
