# Supplementary material for: Conserved Motifs within Hepatitis C Virus Envelope (E2) RNA and Protein Independently Inhibit T Cell Activation
Source: PLoS Pathog. 2015 Sep 30;11(9):e1005183. doi: 10.1371/journal.ppat.1005183 (PMC4589396; doi:10.1371/journal.ppat.1005183)
Supplement: S10 Fig — Representative plots of CD69 surface expression on Jurkat cell lines expressing HCV E2 (384–747) or the Jurkat control cells expressing only GFP (JC) before stimulation and after stimulation with anti-CD3/CD28 or PMA/Ionomycin for 24 hours. Each experiment was repeated at least three times with consistent results. (PDF) [file ppat.1005183.s010.pdf]

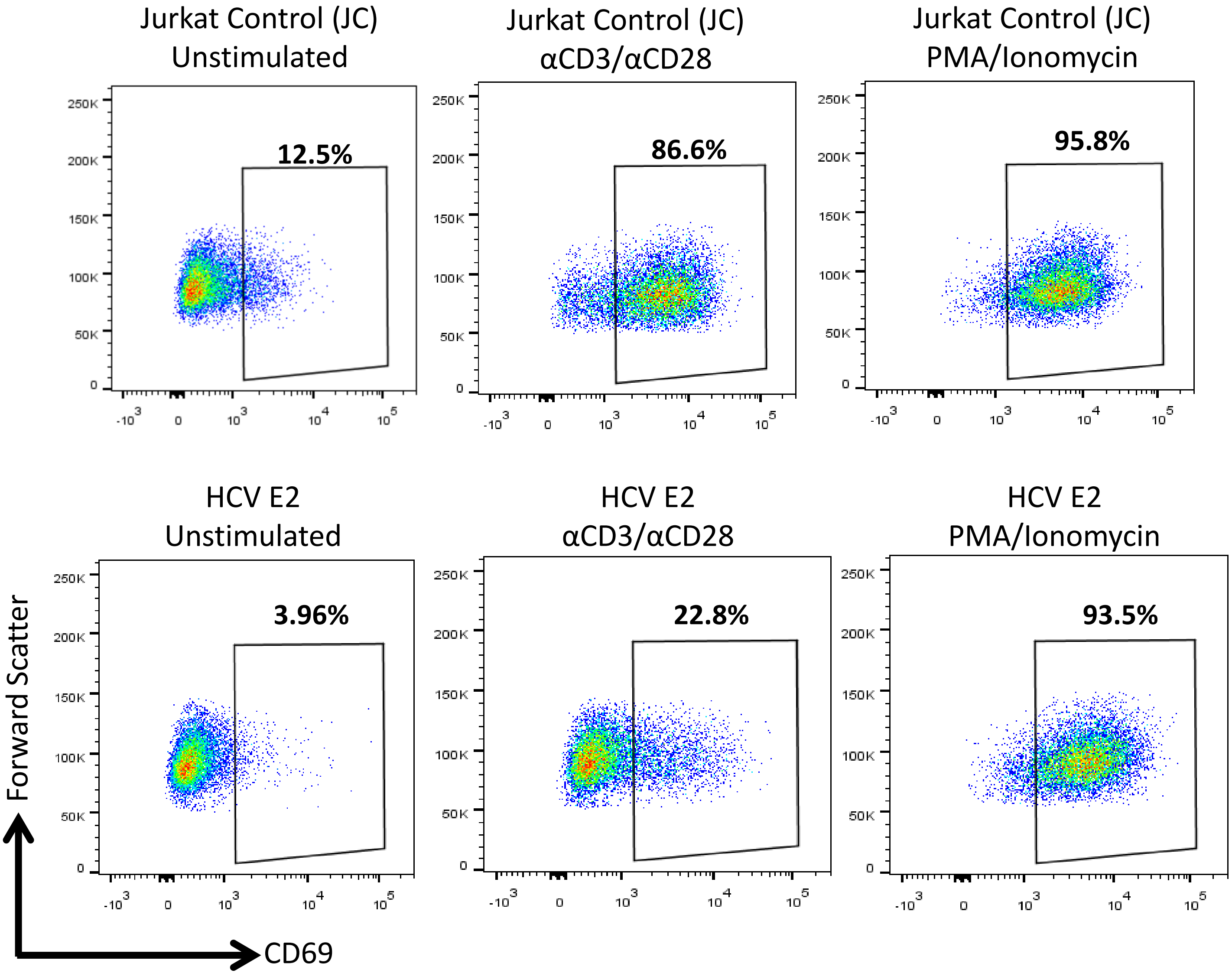

Jurkat Control (JC) Unstimulated
12.5%
Jurkat Control (JC) αCD3/αCD28
86.6%
Jurkat Control (JC) PMA/Ionomycin
95.8%
HCV E2 Unstimulated
3.96%
HCV E2 αCD3/αCD28
22.8%
HCV E2 PMA/Ionomycin
93.5%
250K
200K
150K
100K
50K
0
-10^3
0
10^3
10^4
10^5
Forward Scatter
CD69
